# Nutrient Diagnosis and Precise Fertilization Model Construction of ‘87-1’ Grape (*Vitis vinifera* L.) Cultivated in a Facility

**DOI:** 10.3390/plants14213345

**Published:** 2025-10-31

**Authors:** Haibo Wang, Xiaolong Wang, Chang Liu, Xiangbin Shi, Xiaohao Ji, Shengyuan Wang, Tianzhong Li

**Affiliations:** 1 Laboratory of Fruit Cell and Molecular Breeding, China Agricultural University, Beijing 100193, China; haibo8316@163.com (H.W.); wsy0515@yeah.net (S.W.); 2 The Institute of Pomology, Chinese Academy of Agricultural Sciences, Xingcheng 125100, China; wangxiaolong01@caas.cn (X.W.); liuchang2923@163.com (C.L.); shixiangbin@caas.cn (X.S.); jixiaohao2006@163.com (X.J.)

**Keywords:** formula fertilization, fruit quality, mineral elements, nutritional diagnosis, precise fertilization model

## Abstract

Rape is one of the most widely cultivated and highest-yielding fruit crops in the world. However, research on its precise nutrient diagnosis and fertilization theory is severely lacking, significantly restricting the development of the grape industry. In this study, an L_16_(4^5^) orthogonal experimental design was applied to determine the effects of varying ratios of nitrogen (N), phosphorus (P), potassium (K), calcium (Ca), and magnesium (Mg) on the fruit quality of ‘87-1’ grape (*Vitis vinifera* L.) cultivated in a facility, aiming to optimize nutrient application rates and improve fruit quality. Among the treatments T5 (N2P1K2Ca3Mg4), T14 (N4P2K3Ca1Mg4), and T9 (N3P1K3Ca4Mg2), treatment T9 had the most significant effect on single fruit weight, total soluble solids (TSS) content, fruit firmness (FF), and fruit quality index (FQI) and was conducive to the positive accumulation of the above quality indicators. Based on a comprehensive multi-factor analysis of variance, the optimal fertilization combination for achieving a high FQI was N3P1K2Ca1Mg2, corresponding to application rates of 375.0, 0, 168.8, 0, and 70.5 kg·hm^−2^ for N, P_2_O_5_, K_2_O, CaO, and MgO, respectively. Furthermore, to establish standards for multivariate compositional nutrient diagnosis (CND) and define the nutrient sufficiency range for ‘87-1’ grape fruit cultivated in a facility, the nutrient concentrations in various plant tissues and the soil and the FQI were measured across 80 treatments over five consecutive years. The nutritional status of the grapes cultivated under these treatments was calculated using the Technique for Order Preference by Similarity to Ideal Solution and the CND method. Based on the optimal nutrient ranges for high FQI sub-populations, a precise fertilization model was developed to facilitate economic fertilizer savings, quality improvement, and standardized grape cultivation in a facility.

## 1. Introduction

China has the highest cultivated area and yield of grapes globally, encompassing an area of 726,000 hm^2^ and a yield of 15 million tons; however, the yield and overall quality of these grapes remain suboptimal [1]. This is related to the cultivation technology for grapes, particularly the management techniques for suboptimal fertilization in grape production. Thus, a scientific and reasonable fertilization strategy can significantly enhance grapevine growth, leading to high production quality and increased yield. Nitrogen (N), phosphorus (P), potassium (K), calcium (Ca), and magnesium (Mg) are essential mineral elements that play crucial roles in plant growth and development, and fertilizer application that includes these nutrients can significantly improve fruit tree yield and quality [2,3]. However, within field production systems, the stoichiometric ratios of N, P, K, Ca, and Mg frequently deviate from optimal levels, predominantly due to the absence of scientifically derived and precisely calibrated nutrient ratios. These imbalances disrupt nutrient uptake and assimilation mechanisms in plants, consequently diminishing crop yield and quality, as plants are unable to effectively harness essential elements required for growth, development, and metabolic functions [4]. This also increases the risk of environmental pollution and nutrient loss [5,6]. The application of conventional fertilizers without proper guidance can cause salt accumulation in the soil [7], decreasing the yield and fruit quality of grapevines. Moreover, an appropriate balance of N, P, K, Ca, and Mg can significantly promote plant growth and reduce overall nutrient requirements [8]. For the application of proportion-based fertilizers, it is essential to determine the optimal ratios of N, P, K, Ca, and Mg and appropriate application amounts based on the integration of the fertilizer requirements of the species, the nutrient supply characteristics of the soil, fertilizer efficacy, and the rules governing nutrient interaction [9]. Therefore, determining the patterns of mineral nutrient requirements in grape is fundamental for scientific fertilization.

Insufficient or excessive fertilization negatively impacts grape yield and quality and inhibits or potentially damages vegetative growth [10]. Routine diagnostic assessment of nutritional status forms an essential component of commercial grape production systems. Quantifying nutrient levels in the soil and plants provides a scientific basis for adjusting fertilizer programs, enhancing the precision of nutrient management. Leaf nutritional diagnostic analysis has been extensively utilized for the assessment of the nutritional status of grape orchards in various regions, facilitating more precise management and fertilization practices [11,12]. Compositional nutrient diagnosis (CND) has been used to effectively analyze nutrient levels in grape leaves and to assess the nutritional balance of plants [13]. Furthermore, the CND method, which is based on the concept of nutritional balancing, is more precise in identifying nutritional deficiencies and/or excesses than traditional diagnostic methods [14]. The amounts and proportions of the N, P, K, Ca, and Mg fertilizers applied in China have been substantially influenced by significant variations in climate, soil fertility and texture, and crop varieties. Nutrient diagnosis of the rhizosphere soil for grape is necessary for generating a precise fertilization model that can be used to guide actual production. Therefore, this work evaluated plant and rhizosphere soil nutrition to determine optimal nutrient ranges of plant and soil elements for high-quality grape production by CND and to develop a precise fertilization model combining the fertilizer requirements of the species and nutrient interactions.

In the current study, experiments were performed to determine the mineral nutrient requirements in ‘87-1’ grape (*Vitis vinifera* L.) cultivated in a facility for three consecutive years and in a ‘5416’ field fertilization program for five consecutive years. Through these experiments, this study examines the N, P, K, Ca, and Mg requirements of grapes at each growth stage and formulates soil nutrient diagnostic criteria specific to grapes in Liaoning Province. The precise fertilization model for grapes was developed using a comprehensive evaluation and variance analysis, providing technical support for economic fertilizer savings, quality improvements, and standardized cultivation practices in facility-grown grapes.

## 2. Materials and Methods

### 2.1. Experimental Site

The experiment was performed in the first greenhouse at the “Lazi” mountain of The Institute of Pomology, Chinese Academy of Agricultural Sciences, Xingcheng, Liaoning Province, China, between 120°44′ E and 40°41′ N. The first greenhouse is oriented east–west, with a length of 60 m and a span of 7.5 m. It is located in the Northern Hemisphere, with a warm temperate semi-humid continental climate. The annual average temperature is 8.7 °C, with an annual average daily maximum temperature of 27.9 °C and an annual average daily minimum temperature of −14.0 °C. The average frost-free period spans 175 days, and the average annual sunshine duration is 2792.2 h. In terms of precipitation, the annual amount ranges between 550 and 700 mm, with an average of 603 mm. The base soil was of the skeletal type, with a pH of 6.5–7.0, organic matter content of 1.2%, alkali-hydrolyzed N content of 156.5 mg·kg^−1^, available P content of 683.6 mg·kg^−1^, available K content of 722.7 mg·kg^−1^, exchangeable Ca content of 6.4 mg·g^−1^, and exchangeable Mg content of 849.7 mg·g^−1^.

### 2.2. Plant Material

The ‘87-1’ grape (*Vitis vinifera* L.) is an excellent variety suitable for protected cultivation and an early maturing bud sport of Muscat grape (*Vitis vinifera* L.). Four-year-old ‘87-1’ grape (*Vitis vinifera* L.) plants with similar growth, grafted onto Beta rootstock, were selected as the experimental materials. For the planting configuration, two plants were placed in a single hole, with a row spacing of 1 m × 2 m, and ridge cultivation was adopted, with ridge dimensions of 0.4 m in height, 6.5 m in length, and 0.8 m in width, which represented the primary enrichment area for grapevine roots. The grapevines were trained into a vertical, single-layer, inclined dragon trunk, with a V-shaped leaf curtain and 15 cm shoot spacing. The planting rows were covered with black mulch, and drip irrigation was placed under the mulch for water management. Standard agricultural practices followed those of other field operations.

### 2.3. Experimental Design

#### 2.3.1. Mineral Nutrient Requirements in Each Period

Prior to destructive harvest, 21 fruit trees were selected and numbered. Three trees were collected at each of the following stages: germination (GS), initial flowering (IFS), end bloom (EBS), seed development (SDS), veraison (VS), maturation (MS), and deciduous (DS). Each tree was separated into the roots, trunk, main stem, shoot, leaves, petioles, and inflorescences/fruit, and the N, P, K, Ca, and Mg contents were determined in each tissue. The fruit yield of each tree was determined at MS, and the mineral element content of fruit at MS was included in DS. The experiment was carried out between 2019 and 2021 in three consecutive grape growing seasons. The demand for N, P, K, Ca, and Mg and their ratios were calculated in comparison groups, GS-IFS, IFS-EBS, EBS-SDS, SDS-VS, VS-MS, and MS-DS, according to the following Formulas (1)–(3) [15]:

Mineral element content in the whole plant, g (AWP) = ∑(concentration of mineral element in each tissue × dry mass of the corresponding tissue)(1)

Mineral element demand between any two growth stages, g (DTS) = AWP_later stage_ − AWP_previous stage_ (2)

Mineral element ratio between any two growth stages, % (RTS) = DTS/DTS_GS-DS_ (3)

#### 2.3.2. ‘5416’ Field Fertilization Scheme

The experiment was conducted in a facility, and fertilizers were applied at five doses for each nutrient, namely N, P, K, Ca, and Mg. Each nutrient factor was set at four levels according to the method used by Wang et al. [16], and the experiment adopted the L_16_ (4^5^) orthogonal design. The following fertilizers were used in the experiment: calcium nitrate (11.86% N and 23.73% CaO), potassium nitrate (13.86% N and 46.53% K_2_O), urea (46.67% N), potassium dihydrogen phosphate (52.21% P_2_O_5_ and 34.56% K_2_O), magnesium sulfate (33.33% MgO), calcium acetate (31.79% CaO), potassium sulfate (54.02% K_2_O), and ammonium dihydrogen phosphate (12.17% N and 61.74% P_2_O_5_). All fertilizers were of high chemical purity. The field was fertilized five times in proportion to the requirements of each element throughout the year. The demand ratios of each element in GS-IFS, IFS-EBS, EBS-VS, VS-MS, and MS-DS were N 14:18:52:7:9, P 4:7:54:17:18, K 13:9:60:11:7, Ca 7:7:64:8:14, and Mg 8:6:64:16:6 [17]. The fertilizers were applied to a trench 30 cm away from the trunk parallel to the row where the trees were located, and the trench was 20 cm in width and 20 cm in depth on the side of the drip irrigation tape of the tree. The fertilizers were dissolved in water and covered with soil. There were three replicates per treatment, with ten plants per replicate. The experiment was carried out between 2019 and 2023 in five consecutive grape growing seasons. The experimental design is described in Table 1 and Table 2.

At the full-blossom stage (FBS), VS, and MS, 10–15 inflorescences or fruits, 40–50 leaves (opposite inflorescences or fruit), and 40–50 petioles (opposite inflorescences or spikes), respectively, were collected from each treatment. The tissue samples were rinsed with deionized water; dried in an oven at 105 °C for 20 min; and then dried at 85 °C to a constant mass, crushed, and screened. Soil samples were collected from each treatment at GS, IFS, EBS, VS, and MS at a distance of 30 cm from the trunk and at depths of 0–20 and 20–40 cm on each side of the row. After collection, soil samples were crushed and screened. At MS, fruit was collected from the periphery of trees in each treatment, with 60 fruits harvested from each plant. The samples were transported to the laboratory in an ice bath for storage. Three replicate samples were collected per treatment, with each replicate consisting of three plants.

### 2.4. Determination of Nutrition and Fruit Quality

Nutritional traits, including the N, P, K, Ca, and Mg contents, were analyzed in different tissues at different developmental stages by combined digestion with H_2_SO_4_–H_2_O_2_ [18]. The samples were kept in plastic bags and stored at 4 °C until further analysis. The N content was determined using a total nitrogen analyzer (AMS Alliance F-T1-29 μF, Brand of France, Frépillon, France), and the concentrations of other elements (P, K, Ca, and Mg) were analyzed using an ICP spectrometer (iCAP 6000 Series, Thermo Fisher Scientific, Waltham, MA, USA) [18]. The soil alkali-hydrolyzed N and available P contents were determined using an AMS Alliance analyzer (AMS Alliance F-T1-29 μF, Brand of France, Frépillon, France), and the available K content was determined using a flame spectrophotometer [19]. The exchangeable Ca and Mg contents were determined using an AAS ZEEnit 700 P (Analytik Jena AG, Jena, Germany) [19]. Three replicates were analyzed for each observation.

The single fruit weight (SFW) was determined using a scale with a precision of 1/10,000. The total soluble solids (TSS) content was measured using a handheld refractometer (model PAL-1, Atago, Tokyo, Japan). The fruit firmness (FF) was evaluated using a TA.XT Plus Texture Analyzer (Stable Micro Systems Ltd., Godalming, UK), which performed texture profile analysis [20]. The measurements were recorded using a cylindrical probe placed 1.5 cm away from the blossom scar to assess the fruit shoulder. The experimental design was a randomized complete block setup with three replications for all treatments. The Technique for Order Preference by Similarity to Ideal Solution (TOPSIS) [21] was employed to comprehensively assess the SFW, TSS, and FF of the fruit, which were used to calculate the fruit quality index (FQI). The weights assigned to SFW, TSS, and FF were 0.0470, 0.2089, and 0.1778 [22], respectively.

### 2.5. Determination of the Plant and Soil Nutritional Diagnostic Factor

Plant tissue analysis was used to develop an optimal sampling protocol for ‘87-1’ grapevines under protected cultivation. Based on the nutrient stability across fertilization treatments, we identified a specific tissue at a specific growth stage as the most reliable sampling combination by considering the lowest coefficient of variation for essential nutrients [23]. A tissue and stage were considered stable as a nutritional diagnostic factor when N, P, K, Ca, and Mg fulfilled this rule. Since the mineral element contents in the soil were affected by fertilization, the mineral elements at GS, IFS, EBS, VS, and MS were used as nutritional diagnostic factors. The nutritional diagnostic criteria for plants and soils were established using the CND method [24] based on the standardized FQI. To achieve a high FQI based on a targeted yield, the optimal mineral element content range and a precision fertilization model were determined based on nutritional diagnostic criteria.

### 2.6. Statistical Analysis

Statistical analysis was conducted using Excel 2013 and SPSS 26 software. Analysis of variance (ANOVA) and the least significant difference (LSD) test were used to compare the differences among groups. The F values of different factors at different levels, the theoretical optimum ratio for each quality, and a comprehensive evaluation were performed using SAS 9.4.

## 3. Results

### 3.1. Comprehensive Analysis of the Effect of Formula Fertilization on Grape Fruit Quality

Significant differences were observed in the SFW, TSS, and FF of grapes among fertilization treatments. However, these parameters exhibited high inter-annual variability (Table S1). The changes in rainfall among the years affected the nutrient solubility, nutrient release, and nutrient leaching, potentially affecting agronomic effectiveness. From an annual mean perspective (Table 3), the most obvious effects were observed in T5 for SFW, T14 for TSS, T9 for FF, and T9 for FQI. Specifically, these treatments showed increases of 6.71% (SFW), 10.99% (TSS), 10.30% (FF), and 45.60% (FQI), respectively, compared with treatment T1 (CK). From a factor-level perspective (Table 3), SFW was significantly higher under N level 2 than under level 1. The most obvious effect on the TSS content was observed under N levels 3 and 4, which significantly increased the TSS content by 5.98% and 5.43%, respectively, compared with level 1. The FF was significantly lower under P_2_O_5_ levels 2, 3, and 4 than under level 1, with level 2 showing an FF that was 9.10% lower than that under level 1, which was a significant difference. Range analysis showed that the optimal combinations affecting SFW, TSS content, FF, and FQI were N2P1K1Ca3Mg2, N3P3K3Ca1Mg4, N3P1K2Ca4Mg2, and N3P1K2Ca1Mg2, respectively (Table 3). According to the F value, the order of influence of the five factors on the above-mentioned parameters of grape fruit was as follows: Ca > N > K > P > Mg, N > Ca > K > P > Mg, P > Mg > N > Ca > K, and N > P > Mg > K > Ca, respectively (Table 3).

### 3.2. Plant and Soil Nutrient Diagnosis

Fruit trees acquire nutritional stability once they reach maturity [25], enabling foliar composition diagnostics to inform targeted fertilizer adjustments, which is considered the best way to express balance among plant tissues [26,27]. When considering the optimal tissue and stage for harvest and interpreting the nutritional levels of N, P, K, Ca, and Mg, their stability was confirmed in the petioles at FBS, fruit at MS, fruit at VS, leaves at VS, and petioles at MS, respectively. The coefficients of variation for N, P, K, Ca, and Mg in those tissues and stages were 13.9%, 31.5%, 16.8%, 57.5%, and 50.7%, which were 17.0–58.7%, 8.7–33.9%, 19.8–64.8%, 0.7–46.0%, and 11.5–41.9% lower than the same nutrients in the other tissues and stages, respectively (Table S2).

The functions of the cumulative variance ratios and their FQI cutoff values were determined for samples comprising the five nutrients to classify the high and low FQI values for orchards of ‘87-1’ grapes cultivated in a facility (Table 4 and Table S3). Reference sub-population division was carried out based on the description by Mohammad et al. [24]. Optimum range^1^ and optimum range^2^ (mg·g^−1^) for high FQI values were calculated based on the cutoff value across nutrient contents in plants or soil at each growth stage and the communal cutoff value across nutrient contents in plants and soil at different growth stages, respectively. The cutoff values corresponding to optimum range^1^ in plants and in soil at GS, IFS, EBS, VS, and MS were 0.8151, 0.5193, 0.5118, 0.7450, 0.6336, and 0.6283, respectively (Table 4). These values were applied to determine the high FQI sub-population, and the values for high FQI sub-populations corresponding to those values were 13 (16.25%), 36 (45.00%), 37 (46.25%), 15 (18.75%), 25 (31.25%), and 26 (32.5%). To identify the communal high FQI sub-populations, the cutoff value (0.8151) corresponding to optimum range^2^ was selected. Considering that the nutrient contents in the reference sub-population with a high FQI represent the optimal levels [28], the minimum and maximum values of nutrients in the high FQI reference population were considered the optimum range for ‘87-1’ grape, as shown in Table 4.

### 3.3. Dynamic Analysis of Plant Nutrition in High FQI Sub-Populations

**Figure 1:**
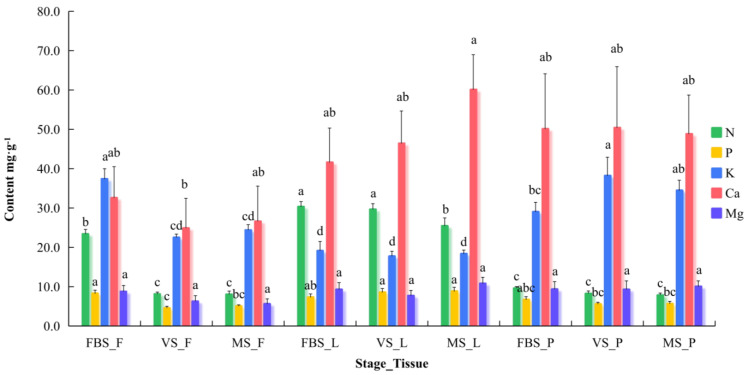
Dynamic analysis of mineral element content in grape plants cultivated in high-quality vineyards. Error bars indicate mean ± SD. Lowercase letters indicate significant differences (*p* < 0.05) in the same element among different stages_tissues. FBS_F, VS_F, and MS_F represent inflorescence of full-blossom stage, fruit of veraison stage, and fruit of maturation stage, respectively. FBS_L, VS_L, and MS_L represent leaf of full-blossom stage, leaf of veraison stage, and leaf of maturation stage, respectively. FBS_P, VS_P, and MS_P represent petiole of full-blossom stage, petiole of veraison stage, and petiole of maturation stage, respectively.

The contents of five mineral elements were quantified in the fruit, leaves, and petioles at three different stages to evaluate the impact of developmental stages on ‘87-1’ grape in high FQI sub-populations (Figure 1 and Table S4). The N, P, and K contents in the inflorescences were significantly higher at FBS (53.2–190.4%) compared with the other two stages. The leaf N content at FBS and VS was significantly higher than that at MS, showing increases of 187.6% and 190.4%, respectively. Compared with FBS, the K content in the petioles was 31.6% higher at VS.

With the exception of the leaf P content at FBS, the N and P contents in the leaves at the three stages were 29.6–215.5%, 53.9–264.8%, and 55.3–222.8% higher than in the other two tissues. The K content in the inflorescences at FBS, petioles at VS, and petioles at MS were 37.3, 38.2, and 34.4 mg·g^−1^, respectively (Table S4), which were significantly higher (28.8–95.4%, 69.6–115.4%, and 41.2–87.6%, respectively) than in the other two tissues at the same stage.

No significant variations were observed in the Ca or Mg contents among tissues or developmental stages. The leaves and petioles showed a higher Ca content, ranging from 41.6 to 60.1 and 48.8 to 50.4 mg·g^−1^, respectively. The Ca content in leaves gradually increased with fruit growth. However, the fruit calcium (Ca) content varied significantly among growth stages, with the highest concentration observed at FBS (32.6 mg·g^−1^), followed by MS (26.6 mg·g^−1^) and VS (24.9 mg·g^−1^) (Table S2). The Mg content in fruit gradually decreased with fruit growth, with the lowest Mg content (5.6 mg·g^−1^) observed at the MS stage (Table S4).

### 3.4. Dynamic Analysis of Soil Nutrition in High FQI Sub-Populations

**Figure 2:**
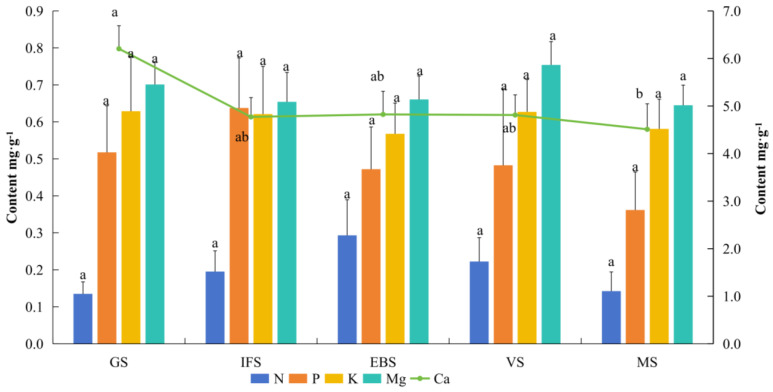
Dynamic analysis of mineral element content in rhizosphere soil cultivated in high-quality vineyards. Error bars indicate mean ± SD. Lowercase letters indicate significant differences (*p* < 0.05) in the same element among different growth stages. GS, IFS, EBS, VS, and MS represent germination stage, initial flowering stage, end bloom stage, veraison stage, and maturation stage, respectively.

Soil nutrients are the fundamental source of essential mineral elements for grape growth and development. Investigating the dynamic patterns in the soil nutrient content across different phenological stages in high FQI sub-populations is necessary to develop precise soil nutrition diagnostic protocols in viticulture. As shown in Figure 2 and Table S5, the variation in mineral nutrients in the soil dynamically changed. The N content in soil was maintained at relatively high levels at EBS but began to decrease after EBS, decreasing 24.2% and 51.4% at VS and MS, respectively. The K and Mg contents showed consistent variation throughout the development stages, i.e., increased 10.5% and 14.1%, respectively, from EBS to VS but declined to 7.3% and 14.5%, respectively, from VS to MS. The P content in the soil peaked at IFS, showing an increase of 23.2–76.3% compared with the other growth stages. The Ca content in the soil peaked at GS (6.2 mg·g^−1^) and remained stable from IFS to MS, with a coefficient of variation of 0.15.

### 3.5. Correlations Among the Mineral Element Contents and Quality Traits

**Figure 3:**
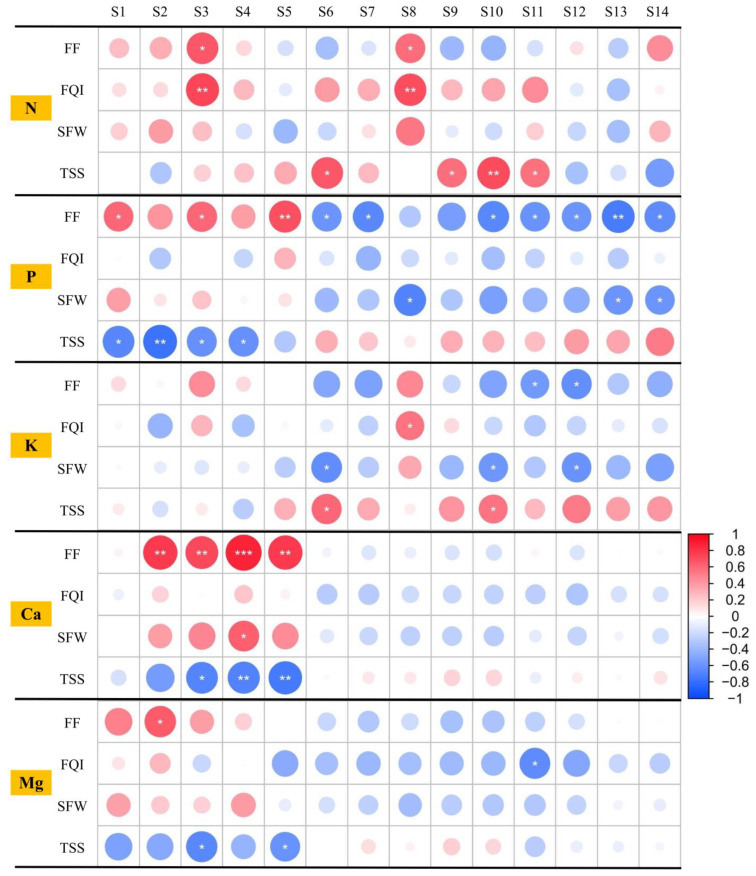
Correlation analysis of mineral element contents and fruit characteristics in high-quality vineyards. FF, QI, SFW, and TSS represent fruit firmness, fruit quality index, single fruit weight, and total soluble solids (TSS) content, respectively. S1, S2, S3, S4, and S5 represent germination stage, initial flowering stage, end bloom stage, veraison stage, and maturation stage, respectively. S6, S7, and S8 represent the inflorescences/fruit of initial flowering, veraison, and maturation, respectively. S9, S10, and S11 represent the leaves of initial flowering, veraison, and maturation, respectively. S12, S13, and S14 represent the petioles of initial flowering, veraison, and maturation, respectively. The symbols *, **, and *** represent the statistical significance of correlation coefficients: * indicates a significant correlation at the *p* < 0.05 level, ** indicates a highly significant correlation at the *p* < 0.01 level, and *** indicates an extremely significant correlation at the *p* < 0.001 level.

The relationships between mineral element contents and quality traits were analyzed using Pearson correlation analysis (Figure 3 and Table S6). SFW was significantly positively correlated with the Ca content in the soil at VS, but it was negatively associated with the P content of MS_F, VS_P, and MS_P and the K content of FBS_F, VS_L, and FBS_P. Significant negative correlations were observed between TSS and P contents at GS and EBS or the Ca content from EBS to MS. Positive correlations were observed between FF and the P content at GS and EBS or the Ca content from EBS to MS. The TSS content was significantly positively correlated with the N content of FBS_F, FBS_L, VS_L, and MS_L and the K content of FBS_F and VS_L. FF had a significant negative correlation with the P content of FBS_F, VS_F, VS_L, MS_L, FBS_P, VS_P, and MS_P and the K content of MS_L and FBS_P. There were significant positive relationships between the FQI and the N content of the soil at EBS and the N and K contents at MS_F. These results suggest that some mineral elements are strongly correlated with quality traits.

### 3.6. Construction and Verification of the Precision Fertilization Model

Based on a tree anatomy experiment of ‘87-1’ grape cultivated in a facility, our analysis established the mineral nutrient demand patterns for grapevines, incorporating both dry matter accumulation dynamics and tissue-specific elemental requirements across developmental stages (Table S8), nutrient concentrations (Table S9), nutrient uptake (Table S10), and nutrient uptake ratio (Table S11) in three consecutive grape growing seasons. On average, the total nutrient uptake was 4.37 kg N, 1.78 kg P, 4.84 kg K, 7.15 kg Ca, and 1.04 kg Mg for every 1000 kg fruit produced (Table S10). The proportion of mineral nutrients required by ‘87-1’ grape cultivated in a facility was 21.8%, 20.1%, 31.6%, 8.8%, and 17.7% for N; 18.2%, 8.3%, 27.1%, 21.6%, and 24.8% for P; 21.9%, 6.7%, 48.4%, 7.1%, and 15.9% for K; 19.7%, 2.9%, 10.3%, 46.7%, and 20.4% for Ca; and 25.2%, 17.7%, 15.7%, 3.8%, and 37.6% for Mg at GS-IFS, IFS-EBS, EBS-VS, VS-MS, and MS-DS, respectively (Table 5 and Table S11). Nutrient uptake in fruit averaged 1.11 kg N, 0.32 kg P, 2.16 kg K, 1.17 kg Ca, and 0.19 kg Mg for every 1000 kg fruit produced (Table S12), with uptake ratios of 25.31%, 17.75%, 44.65%, 16.40%, and 17.95%, respectively, for all tissues.

The precise fertilization model for 1500 kg yield (Table 5) was established using optimum range^2^ (Table 4) and the amount of fertilizer applied (Table 5) at each stage for the high FQI sub-populations. The precision fertilization amount (PFA) under a fixed tree shape was as follows:

PFA (kg) = (1 − FR)(ax + b) + FR(EY/1500)(ax + b), if x < X_max_ (4)

PFA (kg) = 0, if x ≥ X_max_ (5)

Continue to test the nutrient content at the next growth stage; if x of the next growth stage ≥ X_max_, continue to loop Formula (5). If x of the next growth stage < X_max_, continue to loop Formula (4),

Where PFA is the precision fertilization amount of N, P_2_O_5_, K_2_O, CaO, and MgO; FR is the nutrient uptake ratio of fruit to all tissues; a and b are parameters of the fertilization model listed in Table 5; EY is the expected yield (kg); x is the nutrient content of the field to be fertilized; and X_max_ is the maximum value of the optimum range^2^ listed in Table 4.

To verify the reliability of the above-mentioned precision fertilization model, we continued to implement the ‘5416’ field fertilization scheme and two fertilization schemes based on the precision fertilization model in 2024. The amount of fertilizer of T17 and T18 applied at each stage in experimental fields 1 and 2, respectively, based on the precision fertilization model, is listed in Table S13. According to the fertilization recommendations, the soil nutrient contents of N, P, K, Ca, and Mg at each growth stage fall within the optimum range^2^ for the high FQI sub-populations. Based on the FQI, the standardized quality indexes of T18, T17, and T10 were 1.0000, 0.9817, and 0.8655, respectively, which were significantly higher than 0.8151 (Table 6). This shows that T18 and T17 belong to the high FQI sub-population.

## 4. Discussion

The precision application of N, P, K, Ca, and Mg fertilizers effectively ensures nutritional balance in grapevines, improving fruit yield and quality [29,30]. The findings of this experiment demonstrate that balanced N, P, K, Ca, and Mg fertilizer application significantly affects grapevine quality. A comprehensive analysis revealed that N has the most pronounced effect on the TSS content and FQI in ‘87-1’ grapes (Table 3) compared with the other nutrients (P, K, Ca, and Mg), likely due to inherent soil N deficiency. However, excessive N application produced characteristic symptoms, including dark green foliage, reduced fruit set, and delayed maturity, which are typically associated with decreased TSS accumulation and reduced anthocyanin and phenolic compound synthesis [31]. Based on the significant correlations (*p* < 0.05) observed in this experiment, the effects of factor N in FBS_F, FBS_L, VS_L, and MS_L on the TSS content and factor N in MS_F on FQI were positive (Figure 3 and Table S6). The results of these analyses demonstrated a lack of N in the soil in this region for TSS accumulation and FQI in ‘87-1’ grape fruit. N is the most influential element promoting physiological growth, morphology, and fruit production in grapevines due to its integral role as a constituent of various cellular components, such as proteins, chlorophyll, and nucleic acids [32]. Therefore, elevated soil N availability enhances photosynthetic capacity, resulting in greater carbohydrate assimilation, which supports both vegetative growth and fruit maturation processes in grapevines [33]. The TSS content and FQI of ‘87-1’ grape fruit increased then decreased with the increase in N fertilizer application, peaking at fertilization level 3 (Table 3). This indicates that 375 kg·hm^−2^ (Table 1) is an optimal amount of N fertilizer for enhancing the effects on the TSS content and FQI in ‘87-1’ grape.

Except for the TSS content and its components, the mastication characteristics of fruit also play a crucial role in determining their internal quality, which is primarily associated with dietary fiber, particularly pectin [34]. The cell wall structure consists of cellulose, hemicellulose, pectin, and, in some cases, lignin, with pectin playing a key role in hardening the cell wall [35]. During fruit maturation, enzymatic breakdown leads to progressive tissue softening [36]. During this period, cellulase facilitates cellulose and hemicellulose degradation, which improves sweetness and mastication traits of the fruit. Higher P doses positively affected FF [37], which may result from the indirect effects of P metabolism on cellulase accumulation in fruit tissues. In this study, P fertilizer had the most impact on the FF of ‘87-1’ grape fruit, but it is recommended that no additional P fertilizer be applied (Table 3). On average, the total nutrient uptake was 4.37 kg N, 1.78 kg P, 4.84 kg K, 7.15 kg Ca, and 1.04 kg Mg for every 1000 kg fruit of ‘87-1’ grape (Table S12), indicating that it has very low P demands and that the P in the soil can meet this demand. Except for MS_F and FBS_L, the effect of factor P in FBS_F, VS_F, VS_L, MS_L, FBS_P, VS_P, and MS_P on FF was significantly negatively correlated (*p* < 0.05) (Figure 3 and Table S6), demonstrating the sufficiency of P in the soil of this region for the FF of ‘87-1’ grape fruit.

For plants, Ca is an essential element taken directly from the soil [38]. This study shows that Ca is the most uptaken plant nutrient in ‘87-1’ grape cultivated in a facility (Table S12). Ca has essential functions in fruit formation, development, and quality [39]. Fertilization at a rate of 75 kg Ca·ha^−1^ has been shown to increase the yield of wine grapes by approximately 30.92% in Ningxia, China [40]. Our findings indicate that Ca fertilizer had the most significant influence on the SFW of ‘87-1’ grape fruit (Table 3). These results are consistent with those of Ma et al. [41], who investigated the impact of Ca fertilizer on the 100-grain weight and yield of wine grapes. In this experiment, the effects of factor K in FBS_F and VS_L on TSS and factor K in MS_F on FQI were significantly positively correlated (*p* < 0.05) (Figure 3 and Table S6). The soil Mg content at IFS was significantly positively correlated with FF in ‘87-1’ grapes (*p* < 0.05) (Figure 3 and Table S6). These observed relationships between mineral nutrients and quality parameters suggest the important physiological functions of these elements in grapevine development. K serves two critical roles: as a key regulator of photosynthetic efficiency and as an essential cofactor in carbohydrate metabolism and translocation [42]. It promotes cell division and growth by transporting starches and sugars among plant organs [43]. Mg is an essential component of chlorophyll, the key driver of photosynthesis [44].

This study shows that integrated N, P, K, Ca, and Mg application along with their interactions has significant impact on fruit quality and the residual available nutrients in the soil. Fertilizer deficiencies can have negative effects, making it essential to conduct comprehensive analyses of plants and soil to ensure effective fertilization and to support high-quality viticulture practices [45,46]. Analysis of the nutrient composition among tissues provides valuable insights into the nutrients accessible to the plant. The nutrient levels in the leaves [47], petioles [48], inflorescences, and fruit at different developmental stages are compared with a standard, indicating whether specific elements are deficient, adequate, or excessive for maintaining plant performance. The phenological progression of a grapevine determines the necessary nutrients and their quantities, considering the growth of the vegetative and reproductive components of the vines [49,50].

## 5. Conclusions

Our findings demonstrate that chemical fertilization significantly enhanced both plant tissue and soil mineral content, highlighting its efficacy in grape production systems. Based on these results, we recommend adopting precision chemical nutrition practices to optimize the nutrient status and fruit quality of grapevines. This approach aligns with sustainable agriculture principles by enabling targeted fertilizer application that minimizes environmental impacts and maintains productivity. The development of an integrated precision fertilization model is essential for advancing protected grape cultivation, as it addresses three critical objectives: (1) maximizing yield potential, (2) improving fruit quality parameters, and (3) reducing the ecological footprint through efficient nutrient management.

## Figures and Tables

**Table 1:** Test factor level (annual application rate).

Figure	Factor A	Factor B	Factor C	Factor D	Factor E
Level	N (kg·hm^−2^)	P_2_O_5_ (kg·hm^−2^)	K_2_O (kg·hm^−2^)	CaO (kg·hm^−2^)	MgO (kg·hm^−2^)
1 (0)	0	0	0	0	0
2 (1/2)	187.5	70.5	168.8	168.8	70.5
3 (1)	375.0	141.0	337.5	337.5	141.0
4 (3/2)	562.5	211.5	506.25	506.25	211.5

**Table 2:** Treatments of N, P, K, Ca, and Mg with their dosages on grape plants.

Code	Treatment	N (kg·hm^−2^)	P_2_O_5_ (kg·hm^−2^)	K_2_O (kg·hm^−2^)	CaO (kg·hm^−2^)	MgO (kg·hm^−2^)
T1	N1P1K1Ca1Mg1	0	0	0	0	0
T2	N1P2K2Ca2Mg2	0	70.5	168.8	168.8	70.5
T3	N1P3K3Ca3Mg3	0	141.0	337.5	337.5	141.0
T4	N1P4K4Ca4Mg4	0	211.5	506.3	506.3	211.5
T5	N2P1K2Ca3Mg4	187.5	0	168.8	337.5	211.5
T6	N2P2K1Ca4Mg3	187.5	70.5	0	506.3	141.0
T7	N2P3K4Ca1Mg2	187.5	141.0	506.3	0	70.5
T8	N2P4K3Ca2Mg1	187.5	211.5	337.5	168.8	0
T9	N3P1K3Ca4Mg2	375.0	0	337.5	506.3	70.5
T10	N3P2K4Ca3Mg1	375.0	70.5	506.3	337.5	0
T11	N3P3K1Ca2Mg4	375.0	141.0	0	168.8	211.5
T12	N3P4K2Ca1Mg3	375.0	211.5	168.8	0	141.0
T13	N4P1K4Ca2Mg3	562.5	0	506.3	168.8	141.0
T14	N4P2K3Ca1Mg4	562.5	70.5	337.5	0	211.5
T15	N4P3K2Ca4Mg1	562.5	141.0	168.8	506.3	0
T16	N4P4K1Ca3Mg2	562.5	211.5	0	337.5	70.5

The numbers following N, P, K, Ca, and Mg in the second column (Treatment) indicate the test factor level.

**Table 3:** Results and analysis of orthogonal experiment on fruit quality of grape.

Quality	Treatment	Fertilizer Level					SFW (g)	TSS (%)	FF (g)	FQI
		N	P_2_O_5_	K_2_O	CaO	MgO				
	T1	1	1	1	1	1	4.77 ± 0.50a	18.11 ± 1.10a	412.62 ± 25.71ab	0.4123 ± 0.1131a
	T2	1	2	2	2	2	4.62 ± 0.26a	17.85 ± 1.74a	420.17 ± 49.90ab	0.4265 ± 0.2218a
	T3	1	3	3	3	3	4.64 ± 0.49a	18.41 ± 1.01a	399.10 ± 52.60ab	0.4064 ± 0.1478a
	T4	1	4	4	4	4	4.73 ± 0.34a	17.89 ± 1.97a	427.92 ± 62.74ab	0.4491 ± 0.1480a
	T5	2	1	2	3	4	5.09 ± 0.35a	18.63 ± 0.65a	436.88 ± 55.16ab	0.5181 ± 0.1065a
	T6	2	2	1	4	3	4.99 ± 0.51a	18.47 ± 1.11a	381.79 ± 15.14ab	0.3620 ± 0.1234a
	T7	2	3	4	1	2	4.75 ± 0.43a	19.31 ± 1.39a	419.92 ± 61.57ab	0.5221 ± 0.1584a
	T8	2	4	3	2	1	4.86 ± 0.42a	19.26 ± 1.16a	402.00 ± 15.51ab	0.4569 ± 0.1332a
	T9	3	1	3	4	2	4.73 ± 0.78a	18.67 ± 1.23a	455.11 ± 95.64a	0.6003 ± 0.2408a
	T10	3	2	4	3	1	4.83 ± 0.75a	18.79 ± 1.42a	380.28 ± 46.30b	0.3889 ± 0.2002a
	T11	3	3	1	2	4	4.83 ± 0.67a	19.20 ± 1.41a	430.42 ± 44.80ab	0.5435 ± 0.1867a
	T12	3	4	2	1	3	4.54 ± 0.63a	19.90 ± 2.26a	416.74 ± 21.86ab	0.5336 ± 0.1396a
	T13	4	1	4	2	3	4.85 ± 0.48a	18.50 ± 1.60a	414.53 ± 23.05ab	0.4359 ± 0.1383a
	T14	4	2	3	1	4	4.47 ± 0.54a	20.10 ± 2.17a	380.42 ± 66.33b	0.4356 ± 0.1644a
	T15	4	3	2	4	1	4.60 ± 0.48a	19.36 ± 1.46a	404.23 ± 27.20ab	0.4694 ± 0.1614a
	T16	4	4	1	3	2	4.98 ± 0.33a	18.22 ± 1.33a	408.18 ± 34.71ab	0.4176 ± 0.2265a
SFW (g)	L1	4.68 ± 0.10b	4.85 ± 0.17a	4.90 ± 0.12a	4.65 ± 0.17a	4.78 ± 0.13a	Ca > N > K > P > Mg N2P1K1Ca3Mg2			
	L2	4.95 ± 0.13a	4.73 ± 0.22a	4.70 ± 0.27a	4.78 ± 0.13a	4.78 ± 0.17a				
	L3	4.70 ± 0.14b	4.70 ± 0.12a	4.68 ± 0.17a	4.88 ± 0.22a	4.73 ± 0.22a				
	L4	4.73 ± 0.22ab	4.78 ± 0.22a	4.78 ± 0.05a	4.75 ± 0.17a	4.78 ± 0.25a				
	Best level	2	1	1	3	1/2/4				
	F value	0.79	0.33	0.64	0.88	0.01				
TSS (%)	L1	18.06 ± 0.26b	18.48 ± 0.26a	18.50 ± 0.49a	19.35 ± 0.90a	18.88 ± 0.57a	N > Ca > K > P > Mg N3P3K3Ca1Mg4			
	L2	18.92 ± 0.43ab	18.80 ± 0.95a	18.94 ± 0.89a	18.70 ± 0.66a	18.51 ± 0.63a				
	L3	19.14 ± 0.55a	19.07 ± 0.45a	19.11 ± 0.75a	18.51 ± 0.25a	18.82 ± 0.72a				
	L4	19.04 ± 0.85a	18.81 ± 0.93a	18.62 ± 0.59a	18.60 ± 0.61a	18.95 ± 0.93a				
	Best level	3	3	3	1	4				
	F value	2.14	0.52	0.70	1.27	0.32				
FF (g)	L1	414.95 ± 12.27a	429.79 ± 20.16a	408.25 ± 20.09a	407.43 ± 18.25a	399.78 ± 13.78a	P > Mg > N > Ca > K N3P1K2Ca4Mg2			
	L2	410.15 ± 23.67a	390.66 ± 19.68b	419.51 ± 13.46a	416.78 ± 11.85a	425.84 ± 20.30a				
	L3	420.64 ± 31.24a	413.42 ± 14.39ab	409.16 ± 32.09a	406.11 ± 23.58a	403.04 ± 16.20a				
	L4	401.84 ± 14.90a	413.71 ± 11.24ab	410.66 ± 20.99a	417.26 ± 31.49a	418.91 ± 25.94a				
	Best level	3	1	2	4	2				
	F value	0.54	2.19	0.23	0.3	1.32				
FQI	L1	0.4236 ± 0.0190a	0.4916 ± 0.0855a	0.4339 ± 0.0773a	0.4759 ± 0.0609a	0.4319 ± 0.0377a	N > P > Mg > K > Ca N3P1K2Ca1Mg2			
	L2	0.4648 ± 0.0748a	0.4032 ± 0.0341a	0.4869 ± 0.0487a	0.4657 ± 0.0534a	0.4916 ± 0.0865a				
	L3	0.5166 ± 0.0900a	0.4854 ± 0.0612a	0.4748 ± 0.0862a	0.4328 ± 0.0581a	0.4345 ± 0.0727a				
	L4	0.4396 ± 0.0216a	0.4643 ± 0.0492a	0.4490 ± 0.0552a	0.4702 ± 0.0984a	0.4866 ± 0.0524a				
	Best level	3	1	2	1	2				
	F value	1.17	1.15	0.41	0.27	0.74				

The data are presented as the mean and standard deviation (±SD). SFW, TSS, FF, and FQI represent single fruit weight, total soluble solid, fruit firmness, and fruit quality index, respectively. Different letters following numerical values represent the results of inter-group significance analysis at the *p* < 0.05 level.

**Table 4:** The relationship between the cumulative variance of plant and soil elements and the normalized fruit quality index.

Type	Stage_(Tissue) _Element	Functional Relationship	R^2^	A	B	Y = −B/3A	Optimum Range ^1^ (mg·g^−1^)	Optimum Range ^2^ (mg·g^−1^)
Plant	FBS_L_N	F_i^c^_ (V_N_) = −61.412X^3^ + 150.17X^2^ − 191.27X + 105.24	0.9918	−61.412	150.170	0.8151	7.192–12.516	7.192–12.516
	VS_P_P	F_i^c^_ (V_P_) = −269.76X^3^ + 533.91X^2^ − 368.98X + 101.4	0.9504	−269.760	533.910	0.6597	3.097–7.122	3.097–7.122
	FBS_F_K	F_i^c^_ (V_K_) = 123.71X^3^ − 204.39X^2^ − 8.7805X + 99.531	0.9960	123.710	−204.390	0.5507	18.375–27.350	18.375–27.350
	FBS_F_Ca	F_i^c^_ (V_Ca_) = 43.685X^3^ − 50.586X^2^ − 94.536X + 104.11	0.9967	43.685	−50.586	0.3860	23.801–107.960	23.801–107.960
	FBS_F_Mg	F_i^c^_ (V_Mg_) = 110.3X^3^ − 154.03X^2^ − 54.098X + 101.26	0.9975	110.300	−154.030	0.4655	5.166–21.232	5.166–21.232
	R	F_i^c^_ (V_R_) = −49.511X^3^ + 107.22X^2^ − 157.32X + 101.89	0.9920	−49.511	107.220	0.7219		
Soil	GS_N	F_i^c^_ (V_N_) = 28.046X^3^ − 20.587X^2^ − 96.761X + 91.247	0.9941	28.046	−20.587	0.2447	0.017–0.417	0.017–0.336
	GS_P	F_i^c^_ (V_P_) =36.006X^3^ − 23.91X^2^ − 79.894X + 70.59	0.9550	36.006	−23.910	0.2214	0.036–1.542	0.040–1.394
	GS_K	F_i^c^_ (V_K_) = 8.6264X^3^ + 10.172X^2^ − 118.34X + 102.38	0.9943	8.626	10.172	−0.3931	0.126–1.746	0.126–1.746
	GS_Ca	F_i^c^_ (V_Ca_) = 62.153X^3^ − 96.819X^2^ − 59.121X + 94.235	0.9978	62.153	−96.819	0.5193	3.252–9.250	3.530–9.208
	GS_Mg	F_i^c^_ (V_Mg_) = 59.112X^3^ − 38.225X^2^ − 121.36X + 104.55	0.9938	59.112	−38.225	0.2156	0.339–2.382	0.414–1.164
	GS_R	F_i^c^_ (V_R_) = −36.161X^3^ + 96.896X^2^ − 120.17X + 60.185	0.8850	−36.161	96.896	0.8932		
	IFS_N	F_i^c^_ (V_N_) = 164.03X^3^ − 228.51X^2^ − 33.682X + 101.45	0.9979	164.030	−228.510	0.4644	0.012–1.034	0.012–0.590
	IFS_P	F_i^c^_ (V_P_) =3.9675X^3^ + 61.777X^2^ − 166.95X + 107	0.9920	3.968	61.777	−5.1903	0.215–2.111	0.249–2.111
	IFS_K	F_i^c^_ (V_K_) = 40.813X^3^ − 40.888X^2^ − 93.662X + 95.866	0.9965	40.813	−40.888	0.3339	0.105–1.696	0.134–1.592
	IFS_Ca	F_i^c^_ (V_Ca_) = 242.91X^3^ − 373X^2^ + 35.339X + 98.462	0.9966	242.910	−373.000	0.5118	2.702–11.000	2.702–7.418
	IFS_Mg	F_i^c^_ (V_Mg_) = 123.4X^3^ − 174.27X^2^ − 48.309X + 101.6	0.9980	123.400	−174.270	0.4707	0.299–1.886	0.302–1.208
	IFS_R	F_i^c^_ (V_R_) = 78.171X^3^ − 88.892X^2^ − 77.024X + 91.917	0.9934	78.171	−88.892	0.3790		
	EBS_N	F_i^c^_ (V_N_) = 147.11X^3^ − 231.04X^2^ − 8.6896X + 98.115	0.9973	147.110	−231.040	0.5235	0.017–1.160	0.017–1.160
	EBS_P	F_i^c^_ (V_P_) =109.39X^3^ − 170.67X^2^ − 33.997X + 100.97	0.9962	109.390	−170.670	0.5201	0.112–1.591	0.112–1.591
	EBS_K	F_i^c^_ (V_K_) = −18.397X^3^ + 27.472X^2^ − 84.172X + 76.546	0.9736	−18.397	27.472	0.4978	0.148–1.210	0.148–1.210
	EBS_Ca	F_i^c^_ (V_Ca_) = 73.097X^3^ − 111.06X^2^ − 56.813X + 101.48	0.9960	73.097	−111.060	0.5065	2.686–8.473	2.686–8.231
	EBS_Mg	F_i^c^_ (V_Mg_) = −44.971X^3^ + 100.51X^2^ − 146.64X + 95.082	0.9956	−44.971	100.510	0.7450	0.428–1.277	0.428–1.266
	EBS_R	F_i^c^_ (V_R_) = 137.64X_3_ − 205.95X^2^ − 22.919X + 97.99	0.9972	137.640	−205.950	0.4988		
	VS_N	F_i^c^_ (V_N_) = 26.465X^3^ + 4.702X^2^ − 111.76X + 82.782	0.9866	26.465	4.702	−0.0592	0.024–0.966	0.024–0.735
	VS_P	F_i^c^_ (V_P_) =44.319X^3^ − 37.04X^2^ − 94.919X + 94.1	0.9952	44.319	−37.040	0.2786	0.119–4.143	0.119–2.828
	VS_K	F_i^c^_ (VK) = 14.899X^3^ + 10.976X^2^ − 126.11X + 105.48	0.9919	14.899	10.976	−0.2456	0.254–1.212	0.254–1.212
	VS_Ca	F_i^c^_ (V_Ca_) = 133.93X^3^ − 209.96X^2^ − 24.111X + 101.95	0.9974	133.930	−209.960	0.5226	3.043–7.661	3.043–7.095
	VS_Mg	F_i^c^_ (V_Mg_) = −213.05X^3^ + 404.96X^2^ − 266.02X + 71.346	0.8856	−213.050	404.960	0.6336	0.313–1.197	0.477–1.197
	VS_R	F_i^c^_ (V_R_) = −32.596X^3^ + 88.467X^2^ − 123.67X + 70.449	0.9479	−32.596	88.467	0.9047		
	MS_N	F_i^c^_ (V_N_) = 70.275X^3^ − 104.08X^2^ − 51.727X + 85.866	0.9905	70.275	−104.080	0.4937	0.021–0.691	0.021–0.691
	MS_P	F_i^c^_ (V_P_) = 73.229X^3^ − 94.688X^2^ − 69.975X + 101	0.9953	73.229	−94.688	0.4310	0.078–1.258	0.155–1.258
	MS_K	F_i^c^_ (V_K_) = 51.873X^3^ − 44.305X^2^ − 98.387X + 101.85	0.9945	51.873	−44.305	0.2847	0.127–1.448	0.249–1.114
	MS_Ca	F_i^c^_ (V_Ca_) = 31.18X^3^ − 52.216X^2^ − 63.023X + 89.425	0.9942	31.180	−52.216	0.5582	2.659–8.777	2.659–8.777
	MS_Mg	F_i^c^_ (V_Mg_) = −47.682X^3^ + 128.5X^2^ − 165.6X + 86.876	0.9881	−47.682	128.500	0.8983	0.326–1.186	0.391–1.186
	MS_R	F_i^c^_ (V_R_) = −138.77X^3^ + 261.57X^2^ − 166.14X + 41.788	0.5508	−138.770	261.570	0.6283		

Optimum range ^1^ (mg·g^−1^) for high FQI was calculated based on the cutoff value (the bolded data) across nutrient expressions of plant or soil at each growth stage. Optimum range ^2^ (mg·g^−1^) for high FQI was calculated based on the communal cutoff value (0.8151) across nutrient expressions of plant and soil at different growth stages.

**Table 5:** Precise fertilization model and annual requirement ratio of mineral elements.

Stage_Element	Proportion of Annual Absorption (%)	Precise Fertilization Model (y = ax + b)
GS-IFS_N	21.8 ± 2.4b	y = −10.5800x + 3.5549
IFS-EBS_N	20.1 ± 2.2b	y = −9.0830x + 5.3590
EBS-VS_N	31.6 ± 5.7a	y = −5.9055x + 6.8504
VS-MS_N	8.8 ± 3.7c	y = −27.426x + 20.158
MS-DS_N	17.7 ± 3.4b	y = −3.9179x + 2.7073
GS-IFS_P	18.2 ± 0.4c	y = −1.8744x + 2.6130
IFS-EBS_P	8.3 ± 2.0d	y = −0.3029x + 0.6394
EBS-VS_P	27.1 ± 7.8a	y = −0.6673x + 1.0617
VS-MS_P	21.6 ± 2.3b	y = −2.8106x + 7.9485
MS-DS_P	24.8 ± 0.7a	y = −2.1732x + 2.7338
GS-IFS_K	21.9 ± 0.8b	y = −1.4583x + 2.5463
IFS-EBS_K	6.7 ± 1.3c	y = −3.0093x + 4.7907
EBS-VS_K	48.4 ± 6.0a	y = −2.8602x + 3.4608
VS-MS_K	7.1 ± 4.1c	y = −21.138x + 25.619
MS-DS_K	15.9 ± 2.6b	y = −4.2919x + 4.7812
GS-IFS_Ca	19.7 ± 3.1b	y = −0.8322x + 7.6625
IFS-EBS_Ca	2.9 ± 1.3d	y = −0.5010x + 3.7161
EBS-VS_Ca	10.3 ± 2.1c	y = −0.4261x + 3.5069
VS-MS_Ca	46.7 ± 3.2a	y = −5.3307x + 37.821
MS-DS_Ca	20.4 ± 2.9b	y = −0.4413x + 3.8735
GS-IFS_Mg	25.2 ± 1.6b	y = −1.1280x + 1.3130
IFS-EBS_Mg	17.7 ± 3.3c	y = −1.2450x + 1.5040
EBS-VS_Mg	15.7 ± 4.2c	y = −1.0095x + 1.2781
VS-MS_Mg	3.8 ± 2.3d	y = −12.533x + 15.002
MS-DS_Mg	37.6 ± 1.4a	y = −2.8377x + 3.3656

The nitrogen, phosphorus, potassium, calcium, and magnesium of basic fertilizer refer to N, P_2_O_5_, K_2_O, CaO, and MgO, respectively. GS, IFS, EBS, VS, and MS represent germination stage, initial flowering stage, end bloom stage, veraison stage, and maturation stage, respectively. Lowercase letters following numerical values indicate significant differences (*p* < 0.05) in the same element among different growth stages.

**Table 6:** Verification of precision fertilization model.

Treatment	SFW (g)	TSS (%)	FF (g)	FQI	Ranking	Normalized FQI
T1	3.3 ± 0.0cde	17.8 ± 0.1b	493.5 ± 49.4bcd	0.4718	11	0.3925
T2	3.3 ± 0.0cd	17.7 ± 0.1bc	507.3 ± 63.7bcd	0.5080	8	0.4517
T3	3.8 ± 0.1ab	17.1 ± 0.1e	488.2 ± 2.4cd	0.4087	14	0.2894
T4	3.3 ± 0.2de	17.1 ± 0.1de	505.6 ± 35.2bcd	0.4434	12	0.3461
T5	3.3 ± 0.1cd	16.1 ± 0.1g	482.3 ± 49.6cd	0.2527	17	0.0343
T6	3.9 ± 0.3a	17.7 ± 0.7bc	503.9 ± 35.8bcd	0.5182	7	0.4684
T7	3.2 ± 0.3de	16.8 ± 0.1ef	500.3 ± 50.2bcd	0.3882	16	0.2558
T8	3.6 ± 0.1abcd	16.1 ± 0.1g	536.4 ± 36.6abcd	0.4737	10	0.3956
T9	3.2 ± 0.1de	17.7 ± 0.1bc	527.7 ± 44.7abcd	0.5784	5	0.5668
T10	3.6 ± 0.4abcd	17.0 ± 0.1e	606.4 ± 38.3a	0.7611	3	0.8655
T11	2.8 ± 0.3e	16.7 ± 0.1f	447.2 ± 14.4d	0.2317	18	0.0000
T12	3.6 ± 0.1abcd	15.2 ± 0.1h	541.4 ± 41.9abcd	0.4245	13	0.3152
T13	3.3 ± 0.2cde	16.5 ± 0.1f	562.9 ± 78.6abc	0.5923	4	0.5895
T14	3.4 ± 0.2bcd	17.4 ± 0.1cd	507.9 ± 49.3bcd	0.4860	9	0.4157
T15	3.2 ± 0.5de	18.4 ± 0.1a	512.0 ± 74.5bcd	0.5728	6	0.5576
T16	3.6 ± 0.3abcd	17.4 ± 0.1cd	478.3 ± 72.5cd	0.4015	15	0.2776
T17	3.7 ± 0.1abc	18.5 ± 0.1a	572.0 ± 36.4abc	0.8322	2	0.9817
T18	3.6 ± 0.0abcd	17.9 ± 0.2b	586.0 ± 42.2ab	0.8434	1	1.0000

The data are presented as the mean and standard deviation (±SD). SFW, TSS, FF, and FQI represent single fruit weight, total soluble solid, fruit firmness, and fruit quality index, respectively. Lowercase letters following numerical values indicate significant differences (*p* < 0.05) in the same quality among different treatments.

## Data Availability

Data are contained within the article and Supplementary Materials.

## Supplementary Materials

The following supporting information can be downloaded at: https://www.mdpi.com/article/10.3390/plants14213345/s1, Table S1. Annual results and analysis of orthogonal experiment on fruit quality of grape; Table S2. Annual results and analysis of orthogonal experiment on mineral element content (mg·g^−1^) in each tissue of grape; Table S3. Annual results of orthogonal experiment on mineral element content (mg·g^−1^) in rhizosphere soil of grape; Table S4. Annual results of orthogonal experiment on mineral element content (mg·g^−1^) in plant of high FQI subpopulations; Table S5. Annual results of orthogonal experiment on mineral element content (mg·g^−1^) in soil of high FQI subpopulations; Table S6. Correlation coefficients between the mineral element contents (mg·g^−1^) and quality characteristics of ‘87-1’ grape; Table S7. The relationship between the cumulative variance of plant and soil elements and various fruit quality; Table S8. Dry weight (g) of various tissues at different growth stages; Table S9. Nutrient concentration (mg·g^−1^) of various tissues at different growth stages; Table S10. Nutrient uptake (kg) of various growth stages for every 1000 kg of fruit produced; Table S11. Nutrient uptake ratio of various growth stages; Table S12. Nutrient uptake (kg) of various tissues for every 1000 kg of fruit produced; Table S13. Validation of precise fertilization model based on soil nutrient content in experimental field.
