# Supplementary material for: Nutrient Diagnosis and Precise Fertilization Model Construction of ‘87-1’ Grape (Vitis vinifera L.) Cultivated in a Facility
Source: Plants (Basel). 2025 Oct 31;14(21):3345. doi: 10.3390/plants14213345 (PMC12611038; doi:10.3390/plants14213345)
Supplement: Supplementary file 1 [file plants-14-03345-s001.zip › Table S1.pdf]

Table S1. Annual results and analysis of orthogonal experiment on fruit quality of grape

Year	Code	SFW (g)	TSS content (%)	FF (g)	Normalized SFW	Normalized TSS content	Normalized FF	FQI	Normalized FQI	Ranking of FQI
2019	T1	4.4±0.2de	17.9±0.6h	443.1±21.1ab	0.4146	0.0000	0.9182	0.5903	0.6297	10
	T2	4.5±0.3cd	19.2±0.4e	426.5±15.4abc	0.5122	0.2727	0.8281	0.6316	0.7541	6
	T3	5.0±0.0a	18.1±0.4fgh	458.2±27.2a	0.8780	0.0420	1.0000	0.6276	0.7421	7
	T4	4.3±0.1ef	19.7±0.2cde	407.7±15.1abc	0.3415	0.3776	0.7258	0.6093	0.6870	9
	T5	4.8±0.1b	18.7±0.6efgh	418.7±33.6abc	0.7073	0.1678	0.7858	0.5863	0.6177	11
	T6	5.1±0.1a	18.0±0.3gh	373.6±25.7c	0.9268	0.0210	0.5407	0.4248	0.1311	15
	T7	4.7±0.1bc	20.8±0.5b	415.1±19.9abc	0.6341	0.6084	0.7661	0.7132	1.0000	1
	T8	4.2±0.0ef	20.6±1.0bc	393.7±12.8bc	0.2927	0.5734	0.6501	0.6159	0.7068	8
	T9	4.7±0.1bc	19.0±0.2efg	403.5±46.1abc	0.6341	0.2308	0.7030	0.5604	0.5396	13
	T10	4.8±0.1b	19.1±0.9ef	440.4±50.7ab	0.7073	0.2587	0.9032	0.6576	0.8325	5
	T11	5.1±0.0a	21.0±0.5b	404.1±18.2abc	0.9512	0.6503	0.7064	0.6929	0.9388	2
	T12	4.1±0.1f	20.8±0.9b	403.0±10.3abc	0.2195	0.6084	0.7003	0.6587	0.8358	4
	T13	4.2±0.0ef	20.3±0.8bcd	385.8±30.0bc	0.2927	0.4965	0.6073	0.5694	0.5667	12
	T14	3.8±0.1g	22.7±0.5a	273.9±26.4d	0.0000	1.0000	0.0000	0.3813	0.0000	16
	T15	4.2±0.0ef	20.4±0.5bcd	420.6±55.6abc	0.2927	0.5245	0.7958	0.6916	0.9349	3
	T16	5.2±0.1a	19.5±0.3de	373.2±20.0c	1.0000	0.3357	0.5387	0.4928	0.3359	14
2020	T1	4.7±0.1cd	17.6±1.1bcde	373.2±13.5cd	0.6250	0.5820	0.0605	0.3422	0.1492	14
	T2	4.7±0.4cd	18.3±0.5abc	388.3±21.2bcd	0.5833	0.7461	0.1745	0.4435	0.5990	7
	T3	4.5±0.0de	17.0±0.3cde	425.7±12.6bc	0.4167	0.4336	0.4557	0.4491	0.6239	6
	T4	4.7±0.2cd	15.1±0.3f	498.0±29.8a	0.5833	0.0000	1.0000	0.5338	1.0000	1
	T5	4.7±0.2cd	18.0±0.4abcd	383.0±15.1bcd	0.5833	0.6719	0.1346	0.4028	0.4183	9
	T6	4.2±0.1ef	18.6±0.6ab	395.1±28.4bcd	0.2083	0.8242	0.2259	0.4804	0.7629	2
	T7	5.1±0.0ab	17.7±0.6bcd	401.3±27.0bcd	0.9583	0.6133	0.2722	0.4364	0.5675	8
	T8	5.0±0.2abc	18.6±1.0ab	383.1±52.3bcd	0.8333	0.8125	0.1356	0.4583	0.6647	4
	T9	4.7±0.3cd	17.6±0.3bcde	366.5±13.6d	0.6250	0.5820	0.0104	0.3306	0.0977	15
	T10	4.0±0.1f	19.4±0.2a	365.1±40.9d	0.0000	1.0000	0.0000	0.4618	0.6803	3
	T11	4.8±0.2bcd	17.1±0.3bcde	398.1±50.3bcd	0.7083	0.4727	0.2486	0.3583	0.2207	12
	T12	5.2±0.4a	16.2±0.5ef	425.8±13.9bc	1.0000	0.2500	0.4567	0.3925	0.3726	10
	T13	4.5±0.2de	16.7±2.2de	415.6±38.9bcd	0.4583	0.3633	0.3802	0.3775	0.3060	11
	T14	4.5±0.0de	16.7±0.1de	433.3±33.7b	0.4167	0.3750	0.5132	0.4521	0.6372	5
	T15	4.7±0.2cd	17.9±0.5abcd	365.9±7.4d	0.5833	0.6562	0.0062	0.3582	0.2202	13
	T16	5.2±0.1a	16.5±0.7def	397.7±19.1bcd	1.0000	0.3164	0.2450	0.3086	0.0000	16
2021	T1	5.4±0.1ab	19.9±0.3bcde	424.3±19.2def	0.8190	0.6000	0.2669	0.2952	0.2627	8
	T2	5.0±0.3cde	19.0±0.9fg	356.4±23.5h	0.2895	0.2182	0.0000	0.0537	0.0000	16
	T3	5.0±0.5cde	19.2±0.3efg	417.4±6.4def	0.3418	0.2909	0.2396	0.2442	0.2072	12
	T4	5.2±0.3abcd	19.6±0.7cdef	485.1±24.7bc	0.5791	0.4727	0.5062	0.5049	0.4908	4
	T5	5.4±0.2ab	19.6±0.3cdef	493.1±19.0b	0.8029	0.4727	0.5377	0.5358	0.5244	3
	T6	5.3±0.1abc	20.2±0.7abcd	359.4±24.9gh	0.6944	0.7091	0.0118	0.1559	0.1112	14
	T7	4.9±0.1de	20.6±0.2ab	526.1±14.8b	0.1903	0.9091	0.6674	0.6742	0.6750	2
	T8	5.2±0.2abcd	20.4±0.2abc	403.1±31.8defg	0.5550	0.8000	0.1833	0.2492	0.2127	10
	T9	5.3±0.1abc	20.7±0.5ab	610.7±31.4a	0.6300	0.9273	1.0000	0.9730	1.0000	1
	T10	5.4±0.0ab	19.5±0.5def	417.2±22.1def	0.7735	0.4182	0.2390	0.2571	0.2213	9
	T11	5.6±0.2a	19.7±0.1cdef	446.8±28.6cd	1.0000	0.5091	0.3554	0.3702	0.3443	6
	T12	5.2±0.1abcd	19.7±0.4cdef	448.6±20.0cd	0.5402	0.5091	0.3625	0.3729	0.3472	5

	T13	5.1±0.3bcd	20.1±0.3abcd	400.0±3.8efgh	0.4625	0.6909	0.1713	0.2238	0.1850	13
	T14	5.3±0.1abc	20.8±0.3a	392.5±39.8efgh	0.6756	1.0000	0.1417	0.2464	0.2096	11
	T15	4.7±0.1e	20.6±0.5ab	437.9±25.6de	0.0000	0.8909	0.3203	0.3608	0.3341	7
	T16	5.1±0.1bcd	18.5±0.4g	384.9±31.9fgh	0.4464	0.0000	0.1118	0.1133	0.0648	15
2022	T1	4.2±0.2cdefg	17.0±0.6hi	414.0±31.2c	0.3910	0.2857	0.5197	0.3969	0.2354	13
	T2	4.3±0.4cdefg	14.9±0.1j	485.6±44.5ab	0.4079	0.0000	0.9293	0.4560	0.3864	9
	T3	3.9±0.3efgh	18.3±0.1e	323.3±44.3e	0.2040	0.4670	0.0000	0.3047	0.0000	16
	T4	4.8±0.2abc	16.7±0.1i	401.2±36.5cd	0.6728	0.2527	0.4459	0.3561	0.1313	15
	T5	5.4±0.1a	18.1±0.1e	497.9±28.4a	1.0000	0.4396	1.0000	0.6484	0.8777	2
	T6	4.9±0.3abc	17.1±0.2gh	393.9±39.1cd	0.7175	0.3077	0.4042	0.3646	0.1530	14
	T7	4.0±0.4efgh	18.2±0.1e	385.4±18.0cde	0.2913	0.4560	0.3559	0.4051	0.2564	12
	T8	4.7±0.7bcd	18.1±0.1e	425.0±33.0bc	0.6559	0.4396	0.5822	0.5073	0.5174	7
	T9	3.5±0.3h	18.2±0.2e	417.9±45.8bc	0.0000	0.4560	0.5417	0.4775	0.4413	8
	T10	4.3±0.3cdefg	19.7±0.1c	342.5±25.0de	0.4233	0.6648	0.1098	0.4313	0.3233	11
	T11	3.7±0.1gh	19.4±0.1d	400.9±19.3cd	0.1335	0.6154	0.4443	0.5237	0.5592	6
	T12	3.8±0.2fgh	22.2±0.1a	391.8±48.6cd	0.1467	1.0000	0.3926	0.6436	0.8654	3
	T13	5.1±0.2ab	17.6±0.2f	445.6±50.2abc	0.8261	0.3791	0.7006	0.5262	0.5656	5
	T14	4.5±0.6bcde	20.4±0.1b	436.3±50.7abc	0.5099	0.7637	0.6469	0.6963	1.0000	1
	T15	4.1±0.3defg	20.3±0.0b	402.2±22.3cd	0.3206	0.7418	0.4517	0.5919	0.7334	4
	T16	4.4±0.3cdef	17.3±0.0g	425.7±16.2bc	0.4754	0.3297	0.5862	0.4491	0.3687	10
2023	T1	5.1±0.0bcde	18.2±0.2fgh	408.5±68.1bcdef	0.5575	0.4364	0.4354	0.4368	0.4721	8
	T2	4.7±0.1fgh	17.9±0.2gh	444.0±61.8bcd	0.2465	0.3727	0.6493	0.5475	0.6462	5
	T3	4.9±0.5defg	19.6±0.4bc	371.0±14.0def	0.3616	0.7455	0.2096	0.4065	0.4244	10
	T4	4.7±0.0efgh	18.3±0.4fgh	347.7±44.5ef	0.2691	0.4636	0.0691	0.2416	0.1650	15
	T5	5.2±0.1bcd	18.8±0.0def	391.6±15.3bcdef	0.6151	0.5818	0.3338	0.4174	0.4416	9
	T6	5.5±0.4ab	18.5±0.2efg	386.9±66.3cdef	0.8274	0.5000	0.3054	0.3842	0.3893	12
	T7	5.1±0.1cdef	19.2±0.2bcde	371.7±35.8def	0.4930	0.6636	0.2136	0.3818	0.3856	13
	T8	5.2±0.1bcd	18.6±0.3ef	405.1±46.2bcdef	0.6236	0.5364	0.4151	0.4536	0.4985	7
	T9	5.5±0.3ab	17.9±0.1gh	477.0±24.5ab	0.8188	0.3727	0.8482	0.6598	0.8229	3
	T10	5.8±0.2a	16.3±0.4i	336.2±10.0f	1.0000	0.0000	0.0000	0.1367	0.0000	16
	T11	4.9±0.4cdef	18.8±0.8cdef	535.5±84.5a	0.4098	0.5818	1.0000	0.7724	1.0000	1
	T12	4.5±0.2gh	20.7±0.3a	414.5±29.8bcdef	0.1096	1.0000	0.4716	0.6001	0.7290	4
	T13	5.4±0.1bc	17.8±0.3gh	425.6±49.6bcde	0.7115	0.3545	0.5383	0.4826	0.5441	6
	T14	4.3±0.0h	19.8±0.8b	366.2±20.6def	0.0000	0.8091	0.1804	0.4020	0.4173	11
	T15	5.3±0.1bc	17.6±0.1h	394.6±21.3bcdef	0.6633	0.3091	0.3519	0.3443	0.3266	14
	T16	5.1±0.1cdef	19.4±0.2bcd	459.5±35.7abc	0.4992	0.7091	0.7429	0.7244	0.9245	2
