# Supplementary material for: Nutrient Diagnosis and Precise Fertilization Model Construction of ‘87-1’ Grape (Vitis vinifera L.) Cultivated in a Facility
Source: Plants (Basel). 2025 Oct 31;14(21):3345. doi: 10.3390/plants14213345 (PMC12611038; doi:10.3390/plants14213345)
Supplement: Supplementary file 1 [file plants-14-03345-s001.zip › Table S10.pdf]

Table S10. Nutrient uptake (kg) of various growth stages for every 1,000 kg of fruit produced

Year	Stage	N	P	K	Ca	Mg
2019	GS-IFS	0.97	0.30	1.05	1.15	0.26
	IFS-EBS	0.92	0.15	0.26	0.28	0.15
	EBS-SDS	0.83	0.35	1.38	0.42	0.15
	SDS-VS	0.24	0.11	1.15	0.29	0.06
	VS-MS	0.49	0.37	0.19	3.31	0.02
	MS-DS	0.63	0.43	0.61	1.21	0.41
	Total uptake	4.07	1.71	4.65	6.66	1.04
2020	GS-IFS	0.99	0.33	1.15	1.73	0.28
	IFS-EBS	0.83	0.18	0.32	0.13	0.22
	EBS-SDS	0.92	0.32	1.40	0.10	0.06
	SDS-VS	0.56	0.18	0.78	0.50	0.03
	VS-MS	0.42	0.35	0.61	3.22	0.06
	MS-DS	0.71	0.43	0.96	1.77	0.38
	Total uptake	4.43	1.79	5.23	7.45	1.03
2021	GS-IFS	0.88	0.34	0.97	1.36	0.25
	IFS-EBS	0.87	0.11	0.38	0.20	0.18
	EBS-SDS	0.73	0.42	1.40	0.51	0.07
	SDS-VS	0.89	0.07	0.89	0.38	0.12
	VS-MS	0.22	0.44	0.26	3.48	0.04
	MS-DS	1.00	0.46	0.75	1.43	0.39
	Total uptake	4.60	1.85	4.64	7.35	1.05
Average of total uptake		4.37	1.78	4.84	7.15	1.04
