# Supplementary material for: Nutrient Diagnosis and Precise Fertilization Model Construction of ‘87-1’ Grape (Vitis vinifera L.) Cultivated in a Facility
Source: Plants (Basel). 2025 Oct 31;14(21):3345. doi: 10.3390/plants14213345 (PMC12611038; doi:10.3390/plants14213345)
Supplement: Supplementary file 1 [file plants-14-03345-s001.zip › Table S11.pdf]

Table S11. Nutrient uptake ratio of various growth stages

Year	Stage	N (%)	P (%)	K (%)	Ca (%)	Mg (%)
2019	GS-IFS	23.9	17.8	22.7	17.3	24.5
	IFS-EBS	22.6	8.7	5.7	4.2	14.5
	EBS-SDS	20.3	20.6	29.7	6.3	14.7
	SDS-VS	5.9	6.4	24.7	4.3	5.7
	VS-MS	12.0	21.4	4.1	49.6	1.5
	MS-DS	15.4	25.2	13.2	18.2	39.2
2020	GS-IFS	22.4	18.5	21.9	23.2	27.0
	IFS-EBS	18.6	10.1	6.2	1.7	21.0
	EBS-SDS	20.7	17.8	26.7	1.4	6.2
	SDS-VS	12.7	10.2	15.0	6.7	3.2
	VS-MS	9.6	19.4	11.7	43.3	6.0
	MS-DS	16.1	24.0	18.4	23.8	36.6
2021	GS-IFS	19.2	18.3	21.0	18.5	24.2
	IFS-EBS	19.0	6.1	8.2	2.7	17.6
	EBS-SDS	16.0	22.5	30.1	6.9	6.2
	SDS-VS	19.4	3.9	19.1	5.1	11.1
	VS-MS	4.8	24.0	5.5	47.3	4.0
	MS-DS	21.6	25.1	16.1	19.4	37.0
Mean	GS-IFS	21.8	18.2	21.9	19.7	25.2
	IFS-EBS	20.1	8.3	6.7	2.9	17.7
	EBS-VS	31.6	27.1	48.4	10.3	15.7
	VS-MS	8.8	21.6	7.1	46.7	3.8
	MS-DS	17.7	24.8	15.9	20.4	37.6
