# Supplementary material for: Nutrient Diagnosis and Precise Fertilization Model Construction of ‘87-1’ Grape (Vitis vinifera L.) Cultivated in a Facility
Source: Plants (Basel). 2025 Oct 31;14(21):3345. doi: 10.3390/plants14213345 (PMC12611038; doi:10.3390/plants14213345)
Supplement: Supplementary file 1 [file plants-14-03345-s001.zip › Table S12.pdf]

Table S12. Nutrient uptake (kg) of various tissues for every 1,000 kg of fruit produced

Year	Tissue	N	P	K	Ca	Mg
**2019**	Root	0.77	0.41	0.15	0.41	0.06
	Trunk	0.12	0.15	0.13	0.18	0.02
	Main stem	0.23	0.16	0.13	0.42	0.09
	Shoot	1.42	0.51	1.57	2.17	0.39
	Leaf	0.42	0.13	0.32	1.92	0.17
	Petiole	0.10	0.04	0.21	0.37	0.14
	Fruit	1.03	0.31	2.14	1.19	0.18
**2020**	Root	0.98	0.43	0.23	0.51	0.07
	Trunk	0.14	0.14	0.16	0.66	0.02
	Main stem	0.28	0.16	0.30	0.34	0.07
	Shoot	1.35	0.59	1.73	2.61	0.39
	Leaf	0.44	0.13	0.36	1.81	0.18
	Petiole	0.09	0.03	0.20	0.38	0.12
	Fruit	1.14	0.31	2.25	1.13	0.19
**2021**	Root	0.91	0.44	0.16	0.55	0.07
	Trunk	0.16	0.15	0.08	0.51	0.01
	Main stem	0.42	0.22	0.20	0.47	0.08
	Shoot	1.46	0.54	1.56	2.45	0.40
	Leaf	0.41	0.14	0.35	1.80	0.18
	Petiole	0.09	0.04	0.21	0.38	0.13
	Fruit	1.15	0.32	2.08	1.19	0.19
**Mean**	Root	0.89	0.43	0.18	0.49	0.06
	Trunk	0.14	0.15	0.12	0.45	0.01
	Main stem	0.31	0.18	0.21	0.41	0.08
	Shoot	1.41	0.55	1.62	2.41	0.39
	Leaf	0.42	0.13	0.34	1.84	0.18
	Petiole	0.09	0.04	0.21	0.38	0.13
	Fruit	1.11	0.32	2.16	1.17	0.19
