# Supplementary material for: Nutrient Diagnosis and Precise Fertilization Model Construction of ‘87-1’ Grape (Vitis vinifera L.) Cultivated in a Facility
Source: Plants (Basel). 2025 Oct 31;14(21):3345. doi: 10.3390/plants14213345 (PMC12611038; doi:10.3390/plants14213345)
Supplement: Supplementary file 1 [file plants-14-03345-s001.zip › Table S13.pdf]

Table S13. Validation of precise fertilization model based on soil nutrient content in experimental field

Stage_Element	Optimum range[2] ($mg \cdot g^{-1}$)	X_{max}	Fertilization model (y = ax + b)	a	b	Nutrition content[1] ($mg \cdot g^{-1}$)	Amount of fertilizer application[1] (kg)	Nutrition content[2] ($mg \cdot g^{-1}$)	Amount of fertilizer application[2] (kg)
GS_N	0.017 - 0.336	0.3360	y = -10.58x + 3.5549	-10.5800	3.5549	0.158	1.883	0.223	1.196
GS_P	0.040 - 1.394	1.3940	y = -1.8744x + 2.613	-1.8744	2.6130	1.065	0.617	0.869	0.984
GS_K	0.126 - 1.746	1.7460	y = -1.4583x + 2.5463	-1.4583	2.5463	1.384	0.528	1.394	0.513
GS_Ca	3.530 - 9.208	9.2080	y = -0.8322x + 7.6625	-0.8322	7.6625	8.007	0.999	0.452	7.286
GS_Mg	0.414 - 1.164	1.1640	y = -1.128x + 1.313	-1.1280	1.3130	0.756	0.460	1.842	0.000
IFS_N	0.012 - 0.590	0.5900	y = -9.083x + 5.359	-9.0830	5.3590	0.359	2.098	0.702	0.000
IFS_P	0.249 - 2.111	2.1110	y = -0.3029x + 0.6394	-0.3029	0.6394	1.852	0.078	0.529	0.479
IFS_K	0.134 - 1.592	1.5920	y = -3.0093x + 4.7907	-3.0093	4.7907	1.84	0.000	1.002	1.775
IFS_Ca	2.702 - 7.418	7.4180	y = -0.501x + 3.7161	-0.5010	3.7161	6.59	0.415	6.281	0.569
IFS_Mg	0.302 - 1.208	1.2080	y = -1.245x + 1.504	-1.2450	1.5040	1.091	0.146	1.001	0.258
EBS_N	0.017 - 1.160	1.1600	y = -5.9055x + 6.8504	-5.9055	6.8504	0.651	3.006	0.590	3.366
EBS_P	0.112 - 1.591	1.5910	y = -0.6673x + 1.0617	-0.6673	1.0617	1.064	0.352	1.115	0.318
EBS_K	0.148 - 1.210	1.2100	y = -2.8602x + 3.4608	-2.8602	3.4608	0.596	1.756	0.968	0.692
EBS_Ca	2.686 - 8.231	8.2310	y = -0.4261x + 3.5069	-0.4261	3.5069	9.643	0.000	5.698	1.079
EBS_Mg	0.428 - 1.266	1.2660	y = -1.0095x + 1.2781	-1.0095	1.2781	1.055	0.213	1.025	0.243
VS_N	0.024 - 0.735	0.7350	y = -27.426x + 20.158	-27.4260	20.1580	0.866	0.000	0.639	2.633
VS_P	0.119 - 2.828	2.8280	y = -2.8106x + 7.9485	-2.8106	7.9485	1.456	3.856	1.56	3.564
VS_K	0.254 - 1.212	1.2120	y = -21.138x + 25.619	-21.1380	25.6190	0.631	12.281	0.869	7.250
VS_Ca	3.043 - 7.095	7.0950	y = -5.3307x + 37.821	-5.3307	37.8210	5.216	10.016	5.69	7.489
VS_Mg	0.477 - 1.197	1.1970	y = -12.533x + 15.002	-12.5330	15.0020	0.856	4.274	1.056	1.767
MS_N	0.021 - 0.691	0.6910	y = -3.9179x + 2.7073	-3.9179	2.7073	0.590	0.396	0.593	0.384
MS_P	0.155 - 1.258	1.2580	y = -2.1732x + 2.7338	-2.1732	2.7338	0.758	1.087	10.743	0.000
MS_K	0.249 - 1.114	1.1140	y = -4.2919x + 4.7812	-4.2919	4.7812	0.862	1.082	0.895	0.940
MS_Ca	2.659 - 8.777	8.7770	y = -0.4413x + 3.8735	-0.4413	3.8735	5.66	1.376	5.69	1.363
MS_Mg	0.391 - 1.186	1.1860	y = -2.8377x + 3.3656	-2.8377	3.3656	0.598	1.669	0.691	1.405

Note：Nutrition content[1] (mg/g) and Amount of fertilizer application[1] (kg) refer to the parameters of experimental field 1 used to verify the precise fertilization model.

Nutrition content[2] (mg/g) and Amount of fertilizer application[2] (kg) refer to the parameters of experimental field 2 used to verify the precise fertilization model.
