# Supplementary material for: Nutrient Diagnosis and Precise Fertilization Model Construction of ‘87-1’ Grape (Vitis vinifera L.) Cultivated in a Facility
Source: Plants (Basel). 2025 Oct 31;14(21):3345. doi: 10.3390/plants14213345 (PMC12611038; doi:10.3390/plants14213345)
Supplement: Supplementary file 1 [file plants-14-03345-s001.zip › Table S2.pdf]

Table S2. Annual results and analysis of orthogonal experiment on mineral element content (mg·g^{-1}) in each tissue of grape

Tissue	Year	Code	FBS					VS					MS				
			N	P	K	Ca	Mg	N	P	K	Ca	Mg	N	P	K	Ca	Mg
	2019	T1	23.09	10.88	39.58	15.01	6.26	10.34	9.67	26.79	16.19	9.39	4.69	5.51	17.62	6.14	2.35
		T2	23.98	12.15	35.06	15.24	6.55	12.06	10.05	15.04	6.24	1.48	6.98	5.92	23.47	8.41	6.65
		T3	25.35	12.40	42.27	14.84	6.52	12.48	6.32	30.59	9.53	6.56	6.35	5.39	19.42	6.84	2.39
		T4	25.16	11.43	38.68	17.43	5.51	11.79	8.24	29.85	8.07	2.22	6.38	5.70	19.73	8.99	3.71
		T5	26.10	11.24	38.53	14.91	6.69	10.80	6.36	25.29	9.50	3.78	8.47	6.07	23.82	7.46	3.08
		T6	28.20	14.52	34.20	16.86	6.58	11.44	7.20	28.55	10.66	4.32	6.87	5.62	21.47	5.85	3.36
		T7	26.93	12.82	44.13	17.34	13.74	10.44	6.91	27.35	10.44	2.34	5.80	5.53	22.10	5.13	3.16
		T8	22.42	11.55	42.34	17.16	5.99	8.67	5.94	23.14	8.39	2.45	7.95	5.88	25.44	9.83	3.26
		T9	27.11	11.45	40.43	20.82	13.08	10.72	5.32	24.87	11.66	6.22	5.84	4.86	21.21	12.52	6.77
		T10	25.20	11.54	42.40	17.65	5.74	9.23	7.83	23.26	7.24	3.54	6.03	4.97	18.93	5.45	1.71
		T11	26.04	11.06	33.86	14.63	7.92	9.77	6.23	24.33	12.66	11.39	7.63	4.87	21.53	5.27	3.45
		T12	24.99	12.40	34.86	11.57	3.90	9.54	5.23	21.13	7.00	2.62	6.51	5.37	20.50	7.40	2.63
		T13	24.67	9.59	34.28	20.63	10.62	9.79	5.03	21.80	12.83	5.76	6.96	4.96	19.75	11.86	6.97
		T14	27.08	9.24	33.12	18.16	6.32	8.44	4.80	21.09	5.28	1.48	7.08	5.03	18.55	9.67	5.31
		T15	29.23	12.25	36.92	15.43	8.32	9.55	5.37	26.74	10.26	5.82	7.49	7.12	18.34	12.25	6.05
		T16	26.59	12.33	35.36	15.66	5.38	8.81	5.82	21.03	13.07	4.86	6.83	8.40	19.26	5.14	3.77
	2020	T1	11.61	5.78	24.84	52.88	13.99	5.52	4.71	16.62	22.38	6.86	5.26	5.59	16.50	16.83	4.85
		T2	13.74	6.47	25.67	25.51	7.86	5.52	4.27	20.21	22.58	6.88	4.51	5.32	16.32	10.37	2.73
		T3	11.86	6.42	24.03	13.38	3.85	5.46	5.26	21.97	34.92	11.48	4.20	7.48	28.81	29.47	8.29
		T4	13.06	6.51	27.72	35.47	11.12	5.92	3.92	20.01	23.59	7.58	5.26	6.59	19.94	27.07	7.73
		T5	12.45	7.09	28.19	26.95	8.78	4.80	4.41	16.97	19.63	5.08	5.03	5.73	19.71	16.63	5.36
		T6	11.61	6.59	26.66	23.77	7.05	6.59	4.89	20.85	23.34	6.84	4.94	5.92	17.93	18.79	3.35
		T7	13.57	7.19	30.39	17.31	5.49	6.60	6.23	19.69	17.91	5.24	5.43	5.12	18.39	44.63	13.91
		T8	12.89	7.41	31.55	16.18	4.90	5.97	5.00	19.05	22.42	6.63	4.93	3.75	15.67	13.00	3.65
		T9	12.53	7.10	29.14	20.43	5.79	6.37	4.65	20.99	32.29	8.99	5.48	6.94	20.47	33.12	10.08
		T10	15.35	7.02	29.92	11.79	3.23	5.89	4.18	19.28	15.72	3.96	4.75	6.30	17.78	13.91	3.63
		T11	13.14	6.81	26.54	15.18	4.04	4.73	4.07	16.41	14.70	4.31	5.23	6.97	15.98	8.17	2.40
		T12	13.49	7.27	30.95	31.50	10.11	5.87	4.89	20.18	29.72	9.32	5.81	5.79	21.04	25.66	7.31

		T13	13.33	6.80	25.96	12.42	3.72	6.04	5.68	19.58	17.06	4.94	5.85	4.55	15.33	14.93	4.55
		T14	15.09	6.95	33.32	34.56	9.03	7.09	4.84	20.53	27.05	7.98	6.33	4.29	18.91	14.39	4.97
		T15	15.12	7.90	31.59	30.84	8.46	6.68	5.97	19.42	23.77	7.32	5.97	4.39	17.46	33.39	11.01
		T16	14.84	6.84	29.62	31.48	11.02	6.14	4.96	18.59	29.74	8.60	6.02	4.48	17.28	15.99	4.73
		T1	24.52	5.49	26.67	13.52	3.91	8.77	2.63	17.38	7.61	1.78	12.94	3.95	34.01	9.11	2.20
		T2	24.99	5.26	31.91	15.09	4.12	8.68	2.71	21.46	9.12	2.22	12.91	3.24	27.36	5.13	1.37
		T3	23.97	6.05	39.75	18.27	4.51	9.31	2.47	20.90	6.52	1.86	11.08	2.96	25.13	10.43	2.31
		T4	21.29	5.20	28.01	14.78	3.57	9.18	2.90	18.79	5.32	1.31	13.11	3.25	28.84	7.85	1.97
		T5	23.42	5.46	28.26	14.55	4.67	8.83	2.58	22.69	7.08	1.59	11.54	3.72	26.00	33.51	3.45
		T6	18.72	5.70	34.31	15.12	4.00	9.24	2.83	19.62	5.01	1.28	10.23	3.65	34.11	19.98	2.50
		T7	22.20	5.80	33.85	13.25	3.20	8.25	1.97	19.07	13.58	2.47	12.74	3.99	28.67	12.09	1.96
		T8	18.80	5.28	31.76	12.64	3.30	7.61	2.47	16.62	6.79	1.62	10.54	3.39	27.33	34.08	3.47
Fruit	2021	T9	22.85	5.20	32.59	14.93	3.70	8.57	2.05	18.97	7.21	1.73	13.16	4.60	30.72	13.46	2.18
		T10	19.77	5.83	36.00	15.53	3.86	8.59	2.54	22.67	9.98	1.74	9.68	3.01	20.81	9.74	1.42
		T11	21.06	5.67	33.35	13.60	3.22	8.42	2.75	17.80	6.54	1.52	10.52	3.45	25.19	4.61	1.22
		T12	25.27	6.06	35.03	16.45	4.07	10.55	2.84	19.16	8.16	1.98	12.67	4.35	30.89	16.63	2.23
		T13	20.22	5.06	28.14	16.52	4.08	8.55	2.39	18.79	8.13	1.81	9.57	2.70	21.58	8.28	1.29
		T14	18.24	5.21	27.81	10.64	2.72	7.75	2.37	17.28	7.79	1.87	12.88	0.11	1.17	1.14	0.04
		T15	24.41	6.14	35.82	13.14	3.14	7.17	2.43	16.65	4.82	1.15	11.08	3.41	26.36	4.55	1.20
		T16	20.97	5.27	54.94	13.87	3.33	7.08	2.14	16.93	5.95	1.50	11.08	3.41	13.93	22.36	3.30
		T1	20.10	4.94	36.12	77.81	16.11	5.64	5.42	21.87	68.94	13.52	5.08	3.59	19.23	64.50	11.49
		T2	19.36	5.16	39.31	86.87	18.80	4.99	4.09	22.39	73.81	15.29	5.62	3.50	24.62	78.61	16.19
		T3	18.81	6.39	43.28	88.30	19.56	11.09	5.74	28.76	84.09	18.07	6.22	3.58	25.92	68.59	12.58
		T4	19.80	5.97	37.43	81.02	16.96	5.40	4.87	25.27	79.17	17.65	7.97	3.31	26.96	72.57	13.78
		T5	18.83	5.17	36.23	88.88	18.77	5.75	4.72	26.32	76.48	14.31	5.58	3.10	22.52	64.97	11.69
		T6	18.71	5.24	38.26	85.80	18.43	4.67	5.05	23.04	73.10	14.93	5.48	2.12	22.67	74.82	15.17
		T7	20.58	5.81	42.47	84.88	18.47	6.50	4.72	25.90	75.31	15.77	4.99	3.11	22.22	63.74	11.55
	2022	T8	20.30	6.39	47.04	79.18	16.72	6.32	4.76	27.62	76.41	15.48	6.91	3.24	33.03	86.89	17.80
		T9	19.72	5.42	35.29	84.42	17.30	5.81	4.21	25.13	80.52	18.64	5.63	3.30	26.81	80.19	17.64
		T10	20.35	5.59	39.93	78.10	15.78	4.66	4.17	25.09	77.88	17.23	7.29	4.49	27.56	60.37	11.57
		T11	21.20	5.71	38.45	79.32	15.01	5.39	4.31	20.86	66.93	11.88	7.09	5.05	26.19	71.75	14.75
		T12	22.98	5.91	65.34	68.56	12.10	5.54	4.60	23.38	73.82	14.73	5.23	5.09	23.25	68.89	12.70

	T13	18.77	5.84	37.12	85.87	19.92	5.45	4.02	30.71	77.50	15.08	5.26	3.81	26.22	80.71	17.46
	T14	21.27	5.26	36.46	85.78	18.16	5.43	3.88	23.58	65.51	12.20	6.53	6.48	33.67	105.61	15.07
	T15	21.87	6.23	48.57	90.33	18.25	5.01	3.70	27.92	81.94	18.55	5.90	4.48	24.92	71.71	14.48
	T16	21.40	5.90	39.38	74.68	14.80	4.96	3.99	22.52	72.83	14.59	5.96	4.30	24.81	62.86	11.80
	T1	17.18	5.51	27.40	17.41	3.46	8.89	2.72	19.25	11.17	2.28	11.32	3.30	28.19	9.58	2.04
	T2	17.71	4.80	27.47	16.14	2.88	9.70	2.87	22.01	10.79	2.05	8.86	3.30	22.85	9.59	1.93
	T3	19.01	5.04	26.72	17.56	3.54	8.37	2.65	21.06	9.62	2.03	9.12	2.62	23.72	9.43	1.81
	T4	25.78	5.86	30.24	17.43	3.54	7.98	2.23	19.54	9.57	2.02	11.13	3.85	26.35	9.79	2.03
	T5	20.71	5.04	26.20	17.05	2.90	10.18	2.42	22.19	12.50	2.34	8.83	2.72	23.39	6.35	1.54
	T6	17.96	5.18	24.62	14.53	2.72	9.31	2.65	18.85	9.65	2.04	11.50	3.62	28.32	7.03	1.72
	T7	19.20	5.00	24.40	16.51	3.50	8.56	3.67	23.50	11.13	2.17	10.49	3.77	28.50	7.89	1.88
2023	T8	16.63	5.77	31.05	15.25	2.71	8.79	3.18	21.20	9.19	1.63	10.45	3.50	26.46	9.72	2.04
	T9	22.26	5.14	28.37	16.02	2.79	8.75	3.07	19.54	7.69	1.51	11.33	3.48	28.63	9.33	2.25
	T10	19.80	6.58	28.44	17.75	3.33	9.10	2.65	20.04	10.34	1.81	10.35	4.88	25.78	9.45	1.98
	T11	27.84	7.07	33.87	17.30	3.89	8.81	2.73	19.52	9.33	1.88	11.31	4.78	30.42	12.26	2.28
	T12	21.25	6.25	29.71	16.73	3.51	8.41	2.76	16.76	8.78	1.86	8.79	2.88	21.73	11.65	2.25
	T13	20.76	5.66	26.28	16.59	3.27	8.31	2.37	17.75	10.09	2.10	9.31	3.26	21.64	8.51	1.78
	T14	19.38	5.70	27.05	16.60	3.25	8.25	2.78	19.42	9.71	1.93	8.80	3.12	22.57	10.62	1.92
	T15	29.47	7.21	37.23	23.74	4.40	8.09	3.25	20.24	9.70	2.06	10.83	3.46	24.82	7.95	1.84
	T16	22.45	6.68	32.53	19.96	3.85	8.36	2.67	18.38	12.32	2.04	12.81	3.78	26.26	9.21	2.08
**CV (%)**		**22.6**	**34.5**	**21.0**	**85.7**	**69.6**	**25.5**	**40.4**	**16.8**	**103.2**	**87.2**	**33.7**	**31.5**	**23.0**	**104.2**	**87.2**
	T1	33.92	8.89	23.60	20.83	5.33	29.46	13.27	16.78	36.07	8.12	28.01	10.74	19.99	35.86	10.02
	T2	32.86	9.65	14.68	22.86	5.85	30.88	10.34	18.94	32.84	10.31	25.95	11.52	18.94	38.83	9.03
	T3	33.70	7.92	14.23	23.87	8.52	45.59	11.95	18.89	29.71	7.16	25.19	10.39	18.26	36.24	7.82
	T4	34.32	8.32	13.65	24.46	6.76	45.45	10.79	19.14	31.05	6.35	28.18	13.06	17.89	33.15	6.58
	T5	33.08	8.37	15.51	24.32	7.89	45.76	9.62	17.15	28.52	4.83	30.92	12.57	19.19	40.81	7.36
	T6	33.81	11.64	14.78	22.75	8.05	46.11	9.35	14.12	26.37	5.67	33.68	11.95	20.25	38.23	6.81
	T7	33.85	9.72	16.81	22.04	6.12	38.70	9.74	14.59	28.60	4.93	29.74	12.04	19.89	32.86	6.31
2019	T8	32.76	11.20	19.30	24.06	8.05	32.30	11.18	20.21	24.49	6.89	28.89	13.15	21.40	34.23	8.27
	T9	34.16	11.44	17.20	21.45	4.79	31.43	12.84	19.59	23.87	4.35	28.27	12.10	20.91	41.75	13.32
	T10	34.91	11.39	19.61	23.61	7.06	31.86	12.41	20.98	23.80	3.99	28.34	12.37	24.16	43.16	17.27
	T11	35.24	12.10	17.50	28.37	13.51	34.93	13.65	20.17	34.97	11.49	28.95	13.98	19.68	43.10	10.45

		T12	34.58	10.62	17.18	22.53	5.72	33.13	13.85	18.11	24.52	4.85	29.58	14.06	21.83	38.82	18.48
		T13	32.12	11.04	18.43	36.85	10.45	33.23	11.30	22.07	46.53	15.62	30.42	11.91	17.93	36.88	5.34
		T14	33.83	10.47	17.51	22.69	11.81	33.18	12.98	19.15	27.80	8.79	29.92	13.63	23.22	39.77	17.13
		T15	34.90	11.37	44.50	28.80	6.95	34.85	12.42	18.21	33.47	5.97	30.71	13.67	20.59	41.18	8.20
		T16	36.63	11.92	17.46	25.54	5.90	33.91	11.48	16.12	26.48	4.83	28.23	13.36	21.37	42.91	10.00
		T1	22.12	5.81	11.13	29.96	7.47	18.33	5.69	13.06	35.29	7.33	18.90	21.10	11.01	27.11	3.32
		T2	20.93	6.49	14.13	37.56	11.23	20.28	5.26	14.79	33.17	5.96	19.17	7.36	13.45	53.72	10.94
		T3	18.71	6.34	10.09	33.26	8.94	19.59	6.95	12.35	35.67	8.38	21.78	8.48	15.23	72.21	18.43
		T4	19.33	5.74	9.43	32.04	8.91	19.34	8.70	13.58	41.20	8.89	2.75	8.48	15.23	72.21	18.43
		T5	20.03	6.17	9.23	17.86	3.86	17.42	8.14	15.17	66.33	18.58	18.56	7.38	10.87	38.89	7.33
		T6	20.80	5.12	11.15	32.08	7.34	20.17	6.79	17.86	50.32	13.31	19.85	8.21	11.46	30.71	3.33
		T7	21.15	7.47	13.38	22.14	5.36	19.20	7.51	13.99	41.55	10.35	17.72	14.15	14.89	46.02	9.23
	2020	T8	19.33	6.09	12.71	42.01	11.40	17.12	7.33	28.41	58.20	15.25	19.68	12.05	14.56	43.31	8.12
		T9	22.00	6.46	11.20	31.02	7.72	18.86	12.86	16.84	42.19	9.96	19.78	9.23	15.50	46.42	8.02
		T10	19.81	6.22	15.83	53.97	14.39	20.43	11.65	18.52	37.50	7.34	21.39	18.89	17.71	32.52	2.87
		T11	20.22	6.95	12.68	38.73	10.75	17.90	10.80	14.01	41.13	9.69	19.54	7.52	12.53	42.34	8.54
		T12	20.07	6.48	12.80	27.68	6.96	17.80	7.86	14.92	39.24	8.96	25.20	17.62	14.50	32.90	4.77
		T13	19.84	6.52	10.76	37.32	9.62	18.71	11.81	14.08	36.77	7.96	20.98	9.93	15.27	43.65	9.50
		T14	23.09	6.98	13.29	39.67	11.48	19.44	12.02	16.17	39.58	10.16	21.34	5.92	12.68	42.01	8.05
		T15	21.96	11.91	14.18	30.36	6.49	20.56	11.42	11.65	21.78	2.91	22.43	7.33	13.62	42.90	7.87
		T16	21.37	8.43	11.51	38.11	9.85	19.95	12.78	13.35	39.17	8.67	20.39	9.36	12.48	49.64	10.31
		T1	31.33	4.50	12.60	18.05	3.74	25.55	5.08	15.80	31.75	4.14	26.52	4.45	13.51	47.15	5.15
		T2	30.54	4.00	13.93	18.00	3.08	25.17	4.98	14.75	44.53	4.87	28.46	4.21	12.42	35.52	3.92
		T3	29.89	4.42	13.58	18.33	2.82	30.13	4.89	14.62	36.31	4.35	28.23	5.29	13.45	51.00	5.44
		T4	33.45	4.93	12.99	23.84	3.71	29.44	4.36	13.25	23.22	2.71	27.33	4.57	13.46	41.16	4.63
		T5	29.21	3.95	11.41	14.34	2.35	28.11	4.05	12.06	31.16	4.09	27.90	4.78	12.49	49.51	6.80
		T6	29.08	4.92	12.69	22.19	3.56	23.61	3.39	11.26	42.49	4.22	28.21	4.53	12.19	59.57	6.62
		T7	29.93	5.92	15.61	22.36	3.80	28.51	7.31	20.01	36.13	3.91	27.43	6.56	15.22	48.73	5.50
	2021	T8	33.99	6.32	17.10	27.51	4.09	26.23	6.83	14.92	26.73	3.13	30.91	6.14	14.73	42.14	3.97
Leaf		T9	28.83	4.91	21.01	25.52	3.30	30.90	4.87	15.32	29.26	3.08	26.97	5.08	14.50	49.97	4.22
		T10	28.89	4.65	15.91	22.12	3.08	23.82	4.63	15.65	31.20	3.19	27.66	5.35	16.10	41.53	3.98
		T11	31.64	5.74	13.83	20.74	3.45	29.22	4.24	14.36	30.19	3.80	25.63	5.57	12.12	40.17	4.13

	T12	27.65	5.57	13.72	18.60	2.94	29.00	5.87	17.24	32.09	3.73	27.82	6.14	13.75	59.34	5.83
	T13	29.30	4.83	16.52	18.50	3.13	31.64	4.01	20.62	28.00	3.77	26.78	4.61	13.49	39.87	4.76
	T14	29.99	4.56	13.25	32.05	4.12	26.53	5.28	12.76	28.74	3.81	27.40	6.07	14.48	57.47	5.61
	T15	34.25	5.81	19.27	23.87	3.87	27.65	4.22	13.00	29.78	3.26	23.63	4.29	11.30	41.51	3.99
	T16	31.36	5.36	14.19	26.47	4.03	27.31	5.62	27.33	33.89	3.91	27.82	5.56	12.32	47.56	5.40
2022	T1	28.03	4.00	21.20	98.46	20.70	23.84	5.79	18.43	89.19	13.94	18.62	4.88	18.91	122.88	18.45
	T2	26.62	3.68	19.77	93.61	18.99	25.15	6.95	21.81	87.51	13.64	24.94	6.06	23.55	137.23	20.65
	T3	29.47	4.18	16.48	77.41	13.01	28.60	6.06	20.91	97.21	16.72	23.44	6.43	21.76	132.81	20.55
	T4	26.86	4.86	16.76	97.66	19.60	25.55	6.54	23.90	113.57	22.66	25.31	5.65	22.50	137.76	16.94
	T5	26.98	3.94	20.15	96.57	19.46	23.54	5.94	19.25	93.35	14.45	26.76	5.79	19.87	124.56	18.31
	T6	26.91	3.36	15.28	81.51	13.88	27.71	6.91	24.07	114.67	21.00	25.12	6.92	25.44	148.16	17.73
	T7	31.50	4.41	19.45	84.53	14.27	28.83	6.95	23.86	96.27	16.02	25.84	6.35	20.47	121.74	15.83
	T8	29.57	4.69	22.52	79.97	19.50	23.70	5.95	23.16	103.83	17.39	27.11	9.93	27.68	135.50	16.40
	T9	27.94	4.55	20.94	78.63	12.98	23.76	5.29	19.63	94.77	14.97	30.70	10.13	31.01	148.47	17.07
	T10	25.86	5.04	19.58	79.06	12.90	22.36	4.44	21.49	91.51	12.97	23.21	5.24	19.39	103.97	11.30
	T11	28.15	5.13	24.17	102.19	19.81	23.01	6.10	20.35	98.78	14.55	22.52	8.79	22.45	125.35	14.17
	T12	27.37	5.04	20.72	98.31	20.01	27.61	8.90	28.61	107.96	16.39	19.29	5.41	16.34	98.38	11.48
	T13	29.87	3.56	21.23	103.68	21.89	23.86	4.99	19.77	77.64	9.81	26.06	5.33	19.92	106.25	12.73
	T14	26.41	3.94	22.34	94.55	17.92	27.08	5.78	22.23	91.68	14.42	23.46	7.66	22.15	115.82	13.02
	T15	28.64	3.86	19.19	77.82	12.93	25.49	5.52	16.10	74.29	9.67	23.08	5.84	23.88	109.62	12.72
	T16	29.77	5.20	23.75	94.47	19.16	25.55	6.56	24.32	108.59	17.21	23.21	6.40	18.93	104.09	11.91
2023	T1	29.53	4.91	12.42	20.15	3.25	24.55	4.17	12.17	29.03	4.16	30.93	4.33	10.94	40.26	5.17
	T2	26.51	3.92	10.39	19.35	3.00	24.57	3.94	11.81	32.71	3.93	22.78	3.95	13.09	39.00	4.30
	T3	32.33	4.37	11.62	20.22	3.20	26.09	4.87	15.84	39.83	5.07	27.06	3.91	12.98	36.53	4.31
	T4	29.48	5.03	12.65	22.22	3.54	25.72	5.24	14.13	33.98	4.52	24.66	4.95	15.14	38.94	5.19
	T5	31.83	5.15	12.56	24.13	3.74	31.16	4.42	14.77	37.67	4.75	30.62	4.37	13.74	41.06	4.51
	T6	33.87	4.71	12.78	23.57	3.61	25.57	4.67	12.59	27.44	3.53	28.80	4.71	14.51	40.35	4.67
	T7	33.99	5.76	13.51	20.62	3.23	25.85	7.49	14.22	31.90	4.25	31.50	8.37	17.17	48.20	4.63
	T8	27.97	6.07	12.35	25.87	3.64	23.41	7.53	13.33	28.72	3.24	31.48	7.76	15.88	42.14	4.63
	T9	29.01	3.94	12.65	20.95	3.52	26.53	4.76	13.40	30.39	3.46	30.13	4.34	16.06	42.76	4.79
	T10	33.69	5.16	14.08	24.93	4.13	22.94	4.24	13.27	27.69	3.29	26.97	4.11	13.63	42.35	4.38
	T11	33.33	5.86	13.12	24.40	4.35	26.84	4.92	12.90	32.29	4.48	25.93	5.62	13.68	43.35	5.59

	T12	31.48	6.42	13.11	21.76	3.90	30.76	5.81	12.57	33.36	4.66	26.36	7.73	15.53	37.16	4.70
	T13	32.45	4.61	12.43	19.98	3.35	24.38	4.17	15.31	27.97	3.58	22.88	3.71	13.94	37.66	4.34
	T14	27.95	5.08	13.82	22.15	3.93	27.48	4.14	13.28	32.47	4.22	25.87	4.03	14.23	37.64	4.92
	T15	27.23	5.53	14.61	22.94	3.56	24.37	4.46	11.79	30.22	3.58	26.35	4.21	12.13	39.09	4.08
	T16	29.30	6.05	13.35	23.19	3.95	30.22	5.05	12.93	31.92	4.05	27.76	5.63	14.44	34.89	3.80
	**CV (%)**	**16.7**	**38.2**	**31.0**	**69.8**	**67.4**	**23.8**	**41.4**	**24.3**	**57.5**	**62.9**	**17.9**	**47.7**	**25.5**	**57.9**	**57.3**
2019	T1	8.97	8.49	18.36	27.28	10.53	8.25	6.25	23.25	18.74	4.37	5.55	6.77	28.52	25.01	7.23
	T2	7.89	8.51	27.60	22.06	12.16	7.69	6.42	40.04	17.81	3.27	5.93	6.42	34.13	20.93	5.28
	T3	8.98	9.20	26.86	23.70	6.38	9.35	6.39	47.16	17.01	3.16	6.04	6.29	40.33	19.71	4.78
	T4	9.45	9.28	30.58	32.36	14.48	6.57	7.60	32.26	20.92	4.55	6.66	7.92	43.99	24.47	5.56
	T5	10.80	7.83	33.74	20.03	8.97	9.61	6.77	33.15	20.02	4.53	7.47	6.13	41.20	25.68	6.77
	T6	7.89	8.84	22.54	35.06	13.26	9.43	6.63	30.84	22.50	5.15	7.27	6.41	32.06	27.63	6.40
	T7	9.79	9.89	42.85	25.85	8.81	9.53	8.31	48.35	17.48	3.61	6.67	7.91	52.14	21.29	5.34
	T8	8.25	12.08	39.91	22.85	5.96	8.92	9.45	44.27	19.20	3.57	6.23	9.77	44.94	27.51	5.72
	T9	9.76	8.63	39.88	22.10	8.28	7.45	6.74	39.30	19.74	3.28	6.16	6.96	42.45	27.93	6.05
	T10	9.43	9.38	37.17	22.08	5.98	8.66	6.57	38.47	16.89	2.92	6.36	6.98	44.49	24.58	5.17
	T11	9.01	10.35	30.23	26.05	7.43	9.48	7.75	30.29	19.43	5.10	6.54	7.67	28.77	26.37	7.93
	T12	9.92	10.74	36.85	22.89	7.30	11.04	7.12	33.23	17.96	4.68	6.24	9.46	35.84	23.72	6.99
	T13	8.35	8.55	32.13	28.07	10.10	9.16	7.18	37.51	20.21	4.74	5.85	6.70	32.68	26.21	7.66
	T14	9.39	9.42	37.01	21.75	6.96	9.20	6.36	37.01	17.73	4.49	6.61	5.57	29.55	20.89	6.44
	T15	8.97	9.34	25.31	16.74	2.40	11.82	6.59	31.52	17.96	3.91	7.27	7.06	29.70	26.66	6.35
	T16	9.49	8.88	23.61	14.16	2.40	7.86	6.33	27.08	17.90	3.93	6.39	6.64	26.96	24.39	6.13
2020	T1	10.87	7.68	18.69	28.66	6.98	10.66	5.66	24.03	27.00	7.81	11.69	7.38	25.25	36.98	11.46
	T2	11.93	6.79	22.43	30.35	8.40	11.20	5.93	27.94	21.25	5.60	10.66	5.63	29.05	48.26	15.42
	T3	12.31	5.44	21.04	34.90	9.66	10.37	6.52	30.27	28.27	7.57	11.52	5.10	24.62	33.39	9.91
	T4	12.52	5.43	23.38	53.35	15.75	11.74	5.88	27.29	36.15	10.70	10.81	4.31	31.47	37.73	10.82
	T5	11.24	5.79	18.58	36.68	10.06	10.91	6.83	26.57	25.13	7.19	11.10	4.30	26.98	52.53	14.94
	T6	11.56	5.86	19.98	32.21	8.19	11.14	5.56	29.13	24.16	6.79	10.13	5.28	28.34	56.78	14.52
	T7	10.79	6.02	31.14	35.05	9.81	13.23	7.87	32.05	24.93	6.35	10.45	7.05	28.85	40.13	11.64
	T8	11.23	5.64	23.63	33.19	7.61	11.80	6.66	34.33	35.15	9.90	10.51	7.34	30.51	35.15	9.78
	T9	11.43	6.10	23.84	23.76	4.71	11.74	8.16	44.26	27.26	6.73	10.28	6.25	38.40	48.61	13.34
	T10	10.80	5.11	26.14	26.05	6.25	11.46	7.17	42.47	35.38	9.54	10.80	4.98	33.90	39.58	10.64

		T11	10.82	5.80	27.66	33.08	8.82	11.69	6.80	32.08	29.20	8.02	10.70	7.11	52.94	54.92	16.40
		T12	11.80	5.37	28.53	33.55	8.93	11.17	6.16	37.95	48.04	14.78	11.22	6.49	33.07	49.77	14.03
		T13	8.42	2.60	8.30	9.13	1.93	12.03	5.83	29.28	26.63	7.71	11.09	6.56	29.60	47.07	13.50
		T14	10.51	4.89	27.08	40.23	10.84	11.72	5.63	37.10	37.63	11.73	11.30	7.53	34.42	39.42	11.62
		T15	10.98	5.64	23.29	27.74	7.10	11.26	4.23	23.62	27.90	7.50	10.09	7.84	26.61	35.63	9.74
		T16	11.97	5.42	21.37	37.95	10.46	12.14	6.48	36.44	36.31	10.27	10.68	5.32	24.23	41.39	12.33
		T1	11.06	4.72	20.49	19.04	3.55	8.59	3.28	36.70	34.17	5.75	8.00	3.65	26.10	36.77	8.58
		T2	9.82	4.47	21.60	20.45	4.05	7.86	3.14	32.35	21.96	4.91	9.26	3.86	30.93	40.82	9.92
		T3	10.08	4.09	23.84	15.68	2.70	7.82	2.86	33.09	23.24	4.97	8.72	3.61	22.23	36.23	8.42
		T4	9.61	4.11	17.86	15.65	2.99	7.22	3.14	32.03	23.00	5.78	7.25	3.52	27.83	36.13	8.55
		T5	10.24	4.06	24.54	18.38	3.51	7.34	2.54	25.94	41.94	9.45	7.44	2.95	21.94	37.52	9.70
		T6	9.21	4.10	16.10	16.53	3.18	7.37	3.51	30.23	36.81	6.97	8.45	3.15	20.99	38.22	8.81
		T7	10.52	6.02	25.63	17.55	3.10	6.73	5.31	39.74	26.10	5.53	8.58	4.42	25.65	37.74	8.27
		T8	10.11	5.40	22.45	16.61	2.95	7.70	4.33	31.23	23.83	5.36	9.87	4.33	23.26	39.03	7.71
Petiole	2021	T9	9.15	4.48	21.99	17.67	3.32	7.08	3.12	31.90	27.31	5.08	8.53	4.01	30.97	37.77	6.76
		T10	8.74	4.53	21.15	16.93	2.45	7.34	2.83	33.75	23.79	4.65	9.90	3.37	25.08	37.07	6.59
		T11	9.52	5.61	22.49	16.64	2.75	7.24	3.25	25.33	23.14	4.86	9.19	5.24	26.59	42.81	9.23
		T12	10.61	6.17	25.94	66.46	6.67	7.86	4.90	30.39	28.02	6.50	8.54	5.41	24.31	36.40	8.24
		T13	8.93	3.66	22.80	13.63	2.33	7.19	3.27	28.20	26.81	6.11	8.45	3.34	22.50	35.43	8.22
		T14	10.04	4.27	25.68	16.67	2.98	7.23	2.99	30.68	23.92	5.42	9.25	4.47	25.23	35.99	7.74
		T15	8.94	4.38	26.22	47.20	5.33	7.43	3.29	26.66	29.07	5.66	8.18	3.69	20.99	36.88	7.16
		T16	11.46	4.80	23.69	24.85	3.38	6.92	3.86	22.69	27.18	5.80	8.33	5.33	20.06	44.93	8.86
		T1	8.68	2.59	27.98	79.96	11.28	4.88	2.71	42.99	170.61	27.01	6.56	2.62	37.13	124.52	21.86
		T2	9.23	2.66	22.29	81.11	11.58	4.78	2.00	41.06	106.47	17.25	6.24	3.93	51.04	147.10	28.70
		T3	8.90	3.10	27.07	88.58	12.89	4.59	2.97	53.46	158.17	24.11	6.16	3.15	46.58	148.94	22.23
		T4	9.78	3.18	21.99	88.78	13.64	4.90	3.05	47.67	147.80	26.54	6.71	3.27	47.08	161.77	31.03
		T5	8.29	3.22	28.43	144.17	22.39	5.89	2.77	46.19	159.28	23.79	6.16	1.49	28.93	113.76	21.23
		T6	8.17	3.80	24.66	147.20	23.64	7.46	3.94	69.82	217.12	37.04	6.79	2.78	31.44	121.07	22.01
		T7	7.12	3.88	29.98	153.92	24.79	4.94	2.87	54.66	238.54	44.62	6.43	3.43	46.07	120.64	19.91
		T8	9.49	3.91	27.90	133.90	18.99	6.59	4.78	65.57	186.44	33.99	6.14	6.67	53.99	147.42	22.75
	2022	T9	7.63	3.42	34.97	119.30	17.15	5.19	3.28	61.92	110.78	17.89	7.50	4.19	91.82	116.66	17.96
		T10	7.33	2.94	32.34	162.21	23.45	6.56	4.29	70.81	98.11	14.73	13.95	3.19	49.04	113.07	17.46

	T11	8.28	4.66	40.31	168.72	25.88	5.49	13.61	63.07	113.16	18.36	6.18	4.41	40.10	116.57	19.14
	T12	9.69	5.78	39.64	141.15	21.11	6.41	5.53	58.17	94.55	15.12	7.31	6.91	45.78	115.99	18.33
	T13	7.04	3.22	35.82	131.86	15.51	6.21	4.57	148.85	109.80	18.31	5.61	2.55	50.99	112.05	17.60
	T14	8.08	3.89	36.67	125.28	15.77	5.69	6.00	82.06	175.61	27.04	6.06	3.13	46.19	101.21	15.94
	T15	7.75	4.45	42.72	185.07	32.60	5.74	4.07	51.35	167.55	25.63	6.15	5.30	70.53	139.83	20.98
	T16	8.33	5.05	41.15	147.27	23.07	5.30	4.29	50.50	138.49	24.27	7.58	4.75	47.62	120.23	18.88
2023	T1	10.33	5.64	21.88	17.87	3.67	6.45	2.91	29.52	24.22	6.28	7.53	3.08	21.15	36.50	10.44
	T2	8.82	6.31	19.73	21.44	4.20	6.41	2.47	29.61	26.12	5.97	8.03	3.49	30.16	30.92	7.63
	T3	9.11	5.11	21.68	19.70	3.81	6.26	2.45	26.47	27.19	6.50	7.62	3.02	24.63	29.11	7.72
	T4	9.97	5.22	21.00	19.28	3.61	6.14	2.88	31.54	26.98	5.86	9.08	3.44	34.35	30.34	7.89
	T5	10.87	5.28	23.10	19.36	4.06	6.36	2.33	25.72	22.25	5.12	7.04	2.91	23.83	32.94	7.52
	T6	11.40	5.65	20.84	17.49	3.74	7.23	3.91	25.72	29.56	7.37	8.03	2.79	25.81	32.53	7.89
	T7	9.62	6.43	21.64	17.27	3.70	6.36	5.24	25.92	25.15	5.73	9.35	5.68	29.56	31.80	8.40
	T8	12.36	7.25	20.28	21.78	3.87	7.09	4.84	27.23	22.90	5.04	8.73	5.61	25.26	29.40	7.08
	T9	7.19	3.21	17.25	20.26	3.50	7.40	2.77	25.26	25.88	5.60	9.18	3.24	26.07	34.19	7.53
	T10	9.89	3.95	20.24	19.69	3.74	7.59	2.93	30.16	25.72	5.76	8.52	3.46	26.70	32.86	8.58
	T11	12.33	6.32	20.20	18.80	4.38	7.03	4.83	23.94	24.48	6.98	9.95	5.21	23.16	41.61	9.82
	T12	9.72	6.12	18.66	16.71	3.74	6.83	5.08	17.82	24.88	7.97	8.85	5.31	18.22	29.04	10.63
	T13	11.24	4.52	19.04	17.58	3.31	6.36	2.79	27.59	22.65	5.30	9.82	3.40	26.03	26.71	7.24
	T14	8.71	5.71	16.98	17.97	3.39	6.56	2.79	23.84	25.21	6.94	10.49	3.61	23.93	31.17	10.06
	T15	10.36	4.53	20.75	17.01	2.93	6.33	3.90	25.15	24.80	5.02	9.42	3.85	28.12	27.30	6.54
	T16	10.53	4.62	16.90	17.08	3.36	5.88	4.91	19.48	22.38	6.31	11.27	6.13	23.84	29.69	8.40
**CV (%)**		**13.9**	**37.2**	**27.7**	**100.8**	**77.9**	**27.4**	**41.1**	**47.7**	**106.5**	**86.3**	**22.7**	**35.2**	**36.2**	**72.8**	**50.7**
