# Supplementary material for: Nutrient Diagnosis and Precise Fertilization Model Construction of ‘87-1’ Grape (Vitis vinifera L.) Cultivated in a Facility
Source: Plants (Basel). 2025 Oct 31;14(21):3345. doi: 10.3390/plants14213345 (PMC12611038; doi:10.3390/plants14213345)
Supplement: Supplementary file 1 [file plants-14-03345-s001.zip › Table S3.pdf]

Table S3. Annual results of orthogonal experiment on mineral element content ($mg \cdot g^{-1}$) in rhizosphere soil of grape

Stage	Year	Code	N	P	K	Ca	Mg
	2019	T1	0.0241	0.1445	0.3239	3.2523	0.6015
		T2	0.0231	0.1695	0.2950	4.3577	0.5769
		T3	0.0292	0.1148	0.3042	4.7503	0.7293
		T4	0.0243	0.1963	0.4226	4.8846	0.6084
		T5	0.0259	0.0562	0.2516	3.9648	0.6477
		T6	0.0267	0.0743	0.1276	5.0347	0.6676
		T7	0.0261	0.1287	0.2056	3.5301	0.6526
		T8	0.0204	0.1597	0.4108	5.6588	0.5092
		T9	0.0186	0.0486	0.3022	5.9980	0.4642
		T10	0.0166	0.1100	0.4857	6.9691	0.4141
		T11	0.0311	0.0397	0.1259	8.6300	0.7775
		T12	0.0294	0.1002	0.2828	7.4371	0.7357
		T13	0.0210	0.0360	0.4649	7.2506	0.5244
		T14	0.0294	0.1217	0.4147	8.0426	0.7350
		T15	0.0227	0.3360	0.2132	9.2077	0.5665
		T16	0.0232	0.5387	0.1352	6.7969	0.5797
	2020	T1	0.2233	0.7234	1.0712	7.8430	1.5410
		T2	0.0538	1.1133	0.4622	7.1034	1.1771
		T3	0.2113	1.5418	1.2188	8.9463	1.0871
		T4	0.0462	1.3939	0.4125	7.4192	1.1643
		T5	0.0369	0.3991	0.2795	8.0819	1.6212
		T6	0.0922	1.3558	1.3076	9.0947	1.6706
		T7	0.1129	1.3868	1.0903	9.2503	2.3819
		T8	0.0462	0.8675	0.7500	6.5103	1.1580
		T9	0.2013	0.5983	0.8408	7.3324	1.4664
		T10	0.0315	0.3642	0.4311	6.9042	1.2367
		T11	0.0969	0.6595	0.6631	6.1615	1.1115
		T12	0.0456	0.7129	0.4165	8.6300	1.6884
		T13	0.0620	0.6695	1.1263	7.9023	1.7582
		T14	0.0489	0.5191	0.5145	7.6538	1.5726
		T15	0.0568	1.1573	0.6646	8.7490	1.6707
		T16	0.0413	0.8169	0.6514	8.4162	1.8065
GS	2021	T1	0.1449	1.1912	0.7090	6.4102	1.0640
		T2	0.1111	1.0552	0.3768	7.1968	0.8754
		T3	0.1219	1.0728	0.8468	6.9437	0.7751
		T4	0.1646	1.3992	1.2400	7.0886	0.9972
		T5	0.1315	0.8155	0.5005	5.9385	0.7465
		T6	0.1300	1.2505	0.4125	7.7566	0.9278
		T7	0.1182	1.1730	0.9316	6.5527	0.9479
		T8	0.1354	0.9387	0.7790	5.9868	0.8685
		T9	0.1470	0.8287	0.8138	6.6696	0.9434
		T10	0.1789	0.9596	0.9333	5.9779	0.8594
		T11	0.1114	0.8525	0.3401	5.7428	0.9721
		T12	0.2983	1.1963	0.7917	5.5812	0.9725

	T13	0.2903	0.7017	0.8582	6.5489	1.0641
	T14	0.5886	0.9342	0.9237	5.4066	0.8477
	T15	0.3045	1.0695	0.5375	6.1292	0.5744
	T16	0.2894	0.9865	0.5840	6.1756	0.7318
2022	T1	0.2385	0.2763	0.4625	5.5862	0.5788
	T2	0.1774	0.4696	1.3026	6.7922	0.5485
	T3	0.2351	0.7577	1.4039	6.5710	0.5035
	T4	0.2133	0.5302	1.5236	6.4606	0.4893
	T5	0.3319	0.4975	1.7456	6.2490	0.5809
	T6	0.1918	0.6118	1.5462	5.8717	0.4559
	T7	0.1404	0.5468	1.4119	4.1284	0.3803
	T8	0.1062	0.6795	0.9436	5.0312	0.3181
	T9	0.2731	0.3958	0.8134	5.4884	0.2090
	T10	0.8405	0.8126	1.4321	6.3650	0.3355
	T11	0.1738	0.5181	1.1418	4.5416	0.3388
	T12	0.2209	0.3224	1.4458	4.1268	0.4245
	T13	0.1806	0.3583	1.3255	4.3316	0.4183
	T14	0.3363	0.1712	1.3070	4.5946	0.4779
	T15	0.3101	0.1530	1.5386	6.9594	0.6188
	T16	0.1240	0.4432	1.9551	5.1008	0.3023
2023	T1	0.0833	0.6454	0.2635	6.8836	0.8978
	T2	0.0950	0.8321	0.2984	6.5470	0.6819
	T3	0.1030	0.9490	0.4654	7.0475	0.8036
	T4	0.1650	1.1961	0.8372	7.9099	0.8275
	T5	0.3240	0.8171	0.4920	7.9349	1.0314
	T6	0.0926	0.7614	0.2617	6.9987	0.6888
	T7	0.1227	0.9082	1.0697	6.6511	0.8087
	T8	0.1990	1.0246	0.4185	6.9959	0.7751
	T9	0.1471	0.7871	0.4059	5.5610	0.7234
	T10	0.2846	0.7893	1.0935	8.0384	0.8542
	T11	0.2099	0.7215	0.2258	5.8392	0.7018
	T12	0.4168	0.8489	0.5361	5.2702	0.7354
	T13	0.1722	0.7167	0.4275	4.8617	0.8316
	T14	0.2317	0.7727	0.4536	4.5970	0.8783
	T15	0.5234	1.0066	0.5236	5.6139	0.7320
	T16	0.1899	1.2891	0.5032	4.4336	0.9505
2019	T1	0.0293	0.5779	0.1049	3.8960	0.7330
	T2	0.0282	0.5363	0.2666	3.9883	0.7062
	T3	0.0280	0.4778	0.3115	3.7344	0.7008
	T4	0.0244	0.7694	0.4661	11.0002	0.6099
	T5	0.0219	0.3567	0.2320	3.7637	0.5477
	T6	0.0251	0.5330	0.1430	5.6616	0.6268
	T7	0.0233	0.4302	0.3951	3.5466	0.5817
	T8	0.0140	0.4856	0.3814	3.2999	0.3488
	T9	0.0127	0.3383	0.3063	3.0300	0.3179
	T10	0.0121	0.3665	0.4171	2.7017	0.3023
	T11	0.0218	0.3456	0.1344	3.5366	0.5440
	T12	0.0199	0.3763	0.2402	3.1477	0.4973

		T13	0.0167	0.3144	0.4114	3.1400	0.4164
		T14	0.0187	0.3576	0.4141	2.1599	0.4672
		T15	0.0277	0.3574	0.2261	3.7528	0.6922
		T16	0.0315	0.4356	0.1421	4.0029	0.7876
	2020	T1	0.0533	0.3683	0.9816	7.7780	1.5899
		T2	0.0775	1.2125	0.4196	9.7491	1.8856
		T3	0.0248	1.6021	0.3537	9.4730	1.3910
		T4	0.1221	2.1108	0.8285	7.4178	1.2079
		T5	0.0787	0.6541	0.7334	7.0267	1.0902
		T6	0.0747	0.8931	0.4408	7.8658	1.3101
		T7	0.1189	1.5292	0.5487	7.9855	1.3742
		T8	0.1408	1.7953	0.6753	7.9904	1.3787
		T9	0.1462	0.5212	0.7970	7.1956	1.1794
		T10	0.0747	0.7696	0.4418	8.0273	1.3549
		T11	0.0889	0.6982	0.4108	6.4638	1.1128
		T12	0.2167	1.3055	0.4546	6.6368	1.0550
		T13	0.1344	0.4148	0.5872	6.5731	1.0463
		T14	0.2017	0.9236	0.6017	6.4943	1.1504
		T15	0.2637	1.1633	0.7588	8.0823	1.2720
		T16	0.1443	1.4193	0.3455	7.4682	1.0857
**IFS**	2021	T1	0.1231	1.0198	0.6394	7.5989	0.9542
		T2	0.1116	1.1295	0.3371	7.2212	1.1058
		T3	0.1104	1.2913	0.9631	7.8980	0.8816
		T4	0.1285	1.2287	0.6409	8.0369	1.1568
		T5	0.1173	0.7838	0.4528	6.9627	0.6552
		T6	0.2202	0.8674	0.4364	7.7580	0.7910
		T7	0.1263	0.9153	0.3744	7.7323	1.0111
		T8	0.1391	0.9328	0.5463	7.8197	0.9750
		T9	0.1171	0.7322	0.2535	7.0669	1.0522
		T10	0.2102	0.9569	0.4566	6.2548	1.0040
		T11	0.1786	1.0024	1.0946	7.5447	0.9130
		T12	0.1542	0.9891	0.4053	5.9749	0.8511
		T13	0.2114	0.9437	0.5765	6.6520	0.9431
		T14	0.3893	1.0192	0.9161	6.2237	1.0074
		T15	0.1542	1.0780	0.2280	6.8668	0.8710
		T16	0.1851	1.1427	0.2890	7.3741	0.9567
	2022	T1	0.2145	0.3441	1.9457	5.8677	0.4397
		T2	0.1821	0.2179	1.1434	7.9793	0.5135
		T3	0.1449	0.3862	1.0694	6.9729	0.3775
		T4	0.1569	0.3082	1.2895	7.4399	0.4161
		T5	0.2375	0.5015	1.4714	6.1204	0.4133
		T6	0.2265	0.3023	1.3405	6.3578	0.4087
		T7	0.2029	0.3732	1.3198	5.1890	0.3767
		T8	0.1485	0.5483	0.8190	5.5574	0.2986
		T9	0.1509	0.2983	0.9983	4.3346	0.2406
		T10	0.2508	0.4157	1.3846	5.4868	0.3079
		T11	0.0713	0.3837	0.9695	4.5514	0.3444
		T12	0.0918	0.5248	1.5921	4.8312	0.4062

	T13	0.3657	0.4751	0.7998	6.5386	0.5322
	T14	0.3948	0.5123	0.7985	4.5646	0.4565
	T15	0.1073	0.4946	1.6956	6.4549	0.3223
	T16	0.1455	0.2639	1.3139	5.4371	0.3160
2023	T1	0.1028	0.6680	0.1694	4.6704	0.6305
	T2	0.1445	0.8162	0.3244	5.5057	0.7810
	T3	0.0994	0.8125	0.2668	5.1826	0.8108
	T4	0.0984	1.0328	0.2751	6.2842	0.7552
	T5	0.1536	0.7052	0.4912	5.5604	0.7530
	T6	0.1551	0.9417	0.2098	6.2106	0.8241
	T7	0.1285	1.1160	0.7777	4.2698	0.6028
	T8	1.0016	1.1042	1.1804	6.0705	0.8126
	T9	0.4224	0.8449	0.8058	5.0913	0.6241
	T10	0.4325	0.8899	0.9553	4.5469	0.5242
	T11	0.5899	0.9377	0.4478	5.4136	1.0974
	T12	1.0341	0.6395	0.7780	5.0847	0.8484
	T13	0.2904	0.2149	0.8021	4.2502	0.7358
	T14	0.3839	0.2212	0.7375	4.8599	0.7879
	T15	1.1775	0.1941	0.4144	6.5275	0.9532
	T16	0.4558	0.2489	0.4630	4.8377	0.6280
2019	T1	0.0417	0.3956	0.1220	5.5093	1.0422
	T2	0.0214	0.3894	0.1918	3.8309	0.5351
	T3	0.0255	0.4235	0.2861	4.7332	0.6384
	T4	0.0222	0.6413	0.4787	5.6321	0.5553
	T5	0.0186	0.3307	0.2801	3.6601	0.4652
	T6	0.0176	0.3773	0.1670	4.7784	0.4410
	T7	0.0182	0.4597	0.6733	3.2290	0.4558
	T8	0.0209	0.4799	0.4391	3.7103	0.5218
	T9	0.0191	0.2310	0.3495	3.5752	0.4787
	T10	0.0171	0.2853	0.4866	3.2745	0.4280
	T11	0.0324	0.3359	0.1476	3.9456	0.8095
	T12	0.0253	0.3901	0.4401	2.6862	0.6316
	T13	0.0277	0.2476	0.3970	3.9712	0.6913
	T14	0.0293	0.2975	0.4289	3.3328	0.7325
	T15	0.0190	0.2903	0.3840	3.0551	0.4738
	T16	0.0236	0.3006	0.1870	3.4808	0.5891
2020	T1	0.0984	0.4551	0.3298	8.9402	1.7084
	T2	0.0977	1.2224	1.0078	8.2776	1.2441
	T3	0.0466	1.2971	0.7343	7.6185	1.3436
	T4	0.0575	1.5905	0.7878	8.2306	1.2662
	T5	0.1082	0.4034	0.4501	7.5664	1.2108
	T6	0.1208	1.2886	0.4215	8.4729	1.2767
	T7	0.1119	1.0493	0.8630	7.2931	1.1538
	T8	0.1818	1.7012	0.6592	8.5970	1.3982
	T9	0.4231	0.5726	0.7724	7.6770	1.2679
	T10	0.2982	0.9581	0.7481	8.4966	1.4231
	T11	0.3423	0.7159	0.6471	6.5350	1.1528
	T12	0.2624	1.1937	0.7520	7.6428	1.3750

		T13	0.6234	0.4475	0.9081	5.9659	0.9760
		T14	0.2817	0.7337	0.4904	7.2822	1.3377
		T15	0.3059	1.2616	0.4816	8.4476	1.3562
		T16	0.1991	1.6197	0.5650	7.9201	1.3031
**EBS**	2021	T1	0.1463	0.9219	0.3804	7.5708	1.1058
		T2	0.1759	1.2087	1.3509	7.6325	1.1213
		T3	0.1728	1.5303	1.3886	7.9356	0.9831
		T4	0.1510	1.4137	0.6581	8.3540	0.9906
		T5	0.3410	0.8739	0.7812	8.2250	1.1606
		T6	0.4042	1.4510	0.6363	9.6900	1.2290
		T7	0.3958	1.4193	1.5607	7.3205	1.0684
		T8	0.3155	1.4228	0.9849	8.2168	1.0054
		T9	1.1602	1.0739	1.2103	6.5841	0.6929
		T10	0.5386	1.1087	1.2532	6.2677	0.5144
		T11	0.4024	1.0390	0.6164	5.6616	0.6816
		T12	0.4009	1.3827	0.8394	6.1882	0.9315
		T13	0.6495	0.8486	1.1836	6.3005	0.6366
		T14	0.5871	1.0805	0.9408	5.8653	0.5894
		T15	0.5854	1.1257	0.6296	7.2435	0.6335
		T16	0.5192	1.1138	0.3615	6.3185	0.6175
	2022	T1	0.1993	0.4091	0.8619	7.6778	0.6099
		T2	0.1709	0.3825	0.3505	7.8066	0.5659
		T3	0.1773	0.1469	0.4913	7.2608	0.6118
		T4	0.1171	0.4610	0.6222	7.3301	0.5513
		T5	0.2630	0.5472	0.6181	7.5856	0.6702
		T6	0.2972	0.4521	0.5490	8.6611	0.6767
		T7	0.2701	0.7440	0.5098	6.7922	0.6229
		T8	0.3292	0.5284	0.3186	7.3214	0.5813
		T9	0.5078	0.2703	0.6466	5.1877	0.3729
		T10	0.3759	0.2165	0.6113	5.6306	0.4188
		T11	0.4596	0.1376	0.5527	3.2549	0.3345
		T12	0.3707	0.1379	0.9469	4.1237	0.5240
		T13	0.3241	0.2485	0.3763	4.4096	0.4527
		T14	0.7895	0.1122	0.7257	4.8787	0.5868
		T15	0.6015	0.1028	0.7513	5.3998	0.3502
		T16	0.3644	0.1173	0.8511	5.9078	0.4727
	2023	T1	0.1284	0.2051	0.3265	5.2245	0.7543
		T2	0.1614	0.2744	0.5966	6.8608	0.9574
		T3	0.1856	0.3168	0.9935	6.7567	1.0363
		T4	0.1506	0.2646	1.0640	7.0421	0.8880
		T5	0.3835	0.2711	0.7540	6.4657	1.1152
		T6	0.1303	0.2672	0.2007	6.1274	0.8118
		T7	0.1188	0.2629	0.6431	4.7643	0.7970
		T8	0.3799	0.3056	0.6432	5.1294	0.7488
		T9	0.3001	0.2563	0.4173	4.6168	0.6021
		T10	0.4356	0.3145	0.7117	5.2234	0.6537
		T11	0.4185	0.3211	0.2671	5.5759	0.9118
		T12	0.5626	0.3324	0.5687	5.8079	0.9684

		T13	0.2114	0.3223	0.4555	5.4489	0.7300
		T14	0.3275	0.2865	0.4129	4.1802	0.6796
		T15	0.2444	0.3406	0.3648	5.3900	0.7292
		T16	0.3377	0.3342	0.2768	4.9260	0.5363
	2019	T1	0.0258	0.2157	0.1581	4.0045	0.6454
		T2	0.0287	0.2893	0.3708	4.4926	0.7171
		T3	0.0331	0.2646	0.3708	4.7354	0.8268
		T4	0.0305	0.4600	0.6621	5.2534	0.7619
		T5	0.0291	0.1999	0.3242	3.9257	0.7264
		T6	0.0259	0.2786	0.3307	4.8222	0.6477
		T7	0.0270	0.2811	0.7855	3.5024	0.6761
		T8	0.0263	0.2650	0.3996	3.7977	0.6564
		T9	0.0183	0.1618	0.4633	3.5189	0.4568
		T10	0.0236	0.1900	0.4843	3.9529	0.5907
		T11	0.0306	0.1242	0.2620	3.0432	0.7647
		T12	0.0318	0.1980	0.4015	3.0755	0.7938
		T13	0.0346	0.1377	0.7288	3.1284	0.8652
		T14	0.0440	0.1630	0.5238	3.6087	1.0992
		T15	0.0249	0.2133	0.4816	3.8842	0.6214
		T16	0.0327	0.1855	0.1680	3.8486	0.8172
	2020	T1	0.0876	0.7142	0.7063	7.0022	0.9819
		T2	0.0638	1.4938	0.9656	7.0784	1.0335
		T3	0.0181	1.1801	0.3167	6.5826	0.8987
		T4	0.0932	2.8284	1.0753	7.0221	0.9326
		T5	0.0217	0.6214	0.3533	7.5751	1.2255
		T6	0.0351	0.9773	0.2565	7.6606	1.0735
		T7	0.1060	1.7514	1.2421	6.8854	1.1736
		T8	0.1059	4.1430	0.9546	6.5364	0.7575
		T9	0.2080	0.6516	0.8152	7.4558	0.9221
		T10	0.1587	0.7804	0.5127	6.0163	0.9761
		T11	0.2027	0.7702	1.0796	7.1666	0.9415
		T12	0.2013	1.2807	0.6417	6.1379	0.8596
		T13	0.0874	0.5145	0.6924	7.3651	1.0761
		T14	0.5071	0.7182	0.7826	6.0550	0.9518
		T15	0.5252	0.9761	0.8651	8.1870	1.1735
		T16	0.4798	1.0452	0.4302	6.8153	0.8982
**VS**	2021	T1	0.1162	0.7056	0.2358	7.0253	0.6482
		T2	0.1151	0.7844	0.3933	7.3783	0.7082
		T3	0.1205	0.8479	0.6851	7.4104	1.0188
		T4	0.1246	1.2998	0.9606	8.0406	0.7364
		T5	0.1822	1.0573	0.6198	7.3689	0.9220
		T6	0.2160	1.0629	0.4868	7.6086	1.2163
		T7	0.1789	0.9423	1.1911	5.8915	0.7631
		T8	0.2085	1.4400	0.9733	7.3433	0.7905
		T9	0.3547	1.0437	0.6487	7.0953	0.7156
		T10	0.1197	1.3295	0.4073	6.7376	0.6675
		T11	0.4029	1.0954	0.4313	6.3212	0.7315
		T12	0.3637	1.3426	0.5097	7.0469	1.1793

	T13	0.1413	1.0386	0.4597	6.4007	0.5989
	T14	0.3080	0.9581	0.5515	6.4953	0.6699
	T15	0.2670	1.3696	0.8701	6.7154	0.4932
	T16	0.4096	1.1573	0.4054	7.2721	1.1585
2022	T1	0.1646	0.1492	0.5117	6.4316	0.5313
	T2	0.2161	0.2025	0.3305	9.5003	0.8842
	T3	0.1724	0.1915	0.3515	9.5514	0.5620
	T4	0.1527	0.1376	1.2522	4.2048	0.3030
	T5	0.1533	0.1185	1.2120	6.0536	0.4767
	T6	0.1399	0.1419	0.7151	7.2145	0.4357
	T7	0.4403	0.1653	0.5872	4.4421	0.5290
	T8	0.2500	0.1451	0.3249	5.3450	0.4070
	T9	0.3284	0.1153	1.5107	6.4968	0.3567
	T10	0.6242	0.1270	0.6906	6.2317	0.4270
	T11	0.3706	0.2710	0.5564	4.5671	0.5045
	T12	0.5465	0.4663	0.9968	3.5709	0.5309
	T13	0.2904	0.2308	0.5527	3.7232	0.3350
	T14	0.7351	0.2934	0.7310	3.6015	0.5253
	T15	0.9661	0.3609	0.9970	5.7422	0.3132
	T16	0.2141	0.3098	1.0846	3.7141	0.2369
2023	T1	0.1180	0.3314	0.2557	5.7002	0.6399
	T2	0.1211	0.3488	0.6655	5.2936	0.8350
	T3	0.1393	0.3557	0.7052	6.0844	0.8865
	T4	0.1334	0.3581	0.7266	5.4183	0.6180
	T5	0.5407	0.3052	1.0687	7.0272	1.1948
	T6	0.1073	0.2659	0.2080	4.8781	0.5861
	T7	0.1665	0.2237	1.1201	5.2912	0.7628
	T8	0.4716	0.1843	0.7107	5.2539	0.7247
	T9	0.1245	0.1786	0.5089	6.1774	1.1366
	T10	0.2114	0.1561	0.7080	5.7818	0.6437
	T11	0.4136	0.1756	0.2544	5.6199	0.8418
	T12	0.2541	0.1945	0.4829	5.8267	0.8874
	T13	0.1063	0.1552	0.5157	4.5100	0.5524
	T14	0.0843	0.1941	2.2755	5.1936	0.8088
	T15	0.2885	0.1813	0.3468	6.8230	0.9616
	T16	0.3298	0.1609	0.3114	5.9508	1.1971
2019	T1	0.0414	0.0782	0.1274	4.3314	1.0339
	T2	0.0280	0.1831	0.3879	3.6950	0.7005
	T3	0.0323	0.4544	0.5353	6.0572	0.8070
	T4	0.0396	0.3432	1.0059	4.6611	0.9893
	T5	0.0301	0.1187	0.2726	3.2946	0.7528
	T6	0.0375	0.3505	0.1522	5.2210	0.9370
	T7	0.0307	0.2197	0.8122	2.8150	0.7681
	T8	0.0266	0.2788	0.4422	3.0072	0.6648
	T9	0.0261	0.1622	0.4752	3.3834	0.6525
	T10	0.0207	0.1801	0.6124	2.6753	0.5176
	T11	0.0271	0.1550	0.2652	2.7335	0.6776
	T12	0.0301	0.2424	0.4428	2.6589	0.7528

		T13	0.0318	0.1081	0.4961	3.0836	0.7947
		T14	0.0379	0.1354	0.4987	3.0212	0.9478
		T15	0.0269	0.1960	0.3629	3.2846	0.6723
		T16	0.0277	0.1901	0.1775	2.8929	0.6929
	2020	T1	0.0324	0.4063	0.2340	7.5449	0.9754
		T2	0.0677	0.6459	0.7255	7.3661	0.9453
		T3	0.0246	0.6281	0.4175	7.1168	0.9734
		T4	0.0653	1.1178	0.4778	8.7768	1.1864
		T5	0.0380	0.3102	0.3692	6.9032	0.9611
		T6	0.1423	0.6619	0.6166	7.4203	0.8499
		T7	0.1777	0.8084	1.1152	7.9019	1.1558
		T8	0.0874	1.2136	0.6310	7.6816	0.9266
		T9	0.1995	0.4836	0.5480	8.5657	1.0971
		T10	0.2251	0.7230	1.0169	7.6879	0.8055
		T11	0.1167	0.7652	0.3812	7.4157	0.8753
		T12	0.2176	1.0423	0.9077	5.6598	0.5421
		T13	0.3794	0.3800	0.8805	6.0354	0.5080
		T14	0.3733	0.2011	0.6476	6.6420	0.5729
		T15	0.5717	0.3633	0.8165	6.9329	0.5428
		T16	0.5390	0.9013	0.7060	6.6574	0.5973
**MS**	2021	T1	0.1878	1.0709	0.6093	7.0457	1.0502
		T2	0.1234	0.8261	1.5659	7.2993	0.8566
		T3	0.0860	0.9167	0.4291	7.0657	0.8920
		T4	0.1107	1.2049	1.1762	7.8137	0.8510
		T5	0.2639	0.7775	1.0676	7.2865	0.9513
		T6	0.0761	0.9186	0.2735	7.2084	0.8559
		T7	0.1652	0.7226	1.4483	7.7071	1.0092
		T8	0.0885	1.0577	1.2715	6.5782	0.6762
		T9	0.1179	1.2576	0.7665	6.9011	0.5016
		T10	0.2793	0.8205	1.1845	7.5550	0.8672
		T11	0.1450	0.9565	0.3285	6.3804	0.7229
		T12	0.1670	0.9613	0.5154	7.1816	0.9112
		T13	0.1034	0.9317	1.1131	7.0618	0.8730
		T14	0.3054	0.8011	0.8119	6.6757	0.8090
		T15	0.1577	0.7714	0.3907	7.4944	0.8562
		T16	0.1233	1.1950	0.2383	7.7646	1.0056
	2022	T1	0.2059	0.3625	1.0912	6.6491	0.6202
		T2	0.1306	0.4074	0.4228	8.2469	0.8757
		T3	0.1093	0.2778	0.4161	5.7245	0.4399
		T4	0.0992	0.4165	1.1081	7.1458	0.5162
		T5	0.2469	0.1775	0.9081	5.7855	0.5571
		T6	0.0917	0.2039	0.5806	6.4576	0.4663
		T7	0.2170	0.2525	0.7840	4.8081	0.5200
		T8	0.1041	0.5923	0.3523	6.0576	0.3706
		T9	0.1148	0.1836	1.3884	6.0126	0.3204
		T10	0.2030	0.3057	1.0868	4.9176	0.2250
		T11	0.1171	0.2284	0.8587	4.0410	0.3980
		T12	0.6907	0.3344	1.1135	3.6140	0.5057

	T13	0.1844	0.1611	1.2094	3.5498	0.3211
	T14	0.3081	0.2494	0.8994	3.2988	0.3911
	T15	0.5504	0.3413	0.6149	5.5576	0.3263
	T16	0.2755	0.2698	0.3028	4.6328	0.3160
2023	T1	0.0898	0.1845	0.1393	5.6065	0.9506
	T2	0.0910	0.1911	0.3322	6.2015	1.0223
	T3	0.0862	0.1923	0.2876	6.5363	1.4802
	T4	0.0894	0.1919	0.5725	6.3324	0.8300
	T5	0.0980	0.1651	0.3751	6.0217	0.6969
	T6	0.0880	0.1748	0.1655	5.5573	0.6506
	T7	0.0731	0.1514	0.9036	4.5979	0.6416
	T8	0.0745	0.1710	0.2739	5.0118	0.6374
	T9	0.1075	0.1707	0.3605	5.6561	0.5916
	T10	0.0934	0.1809	0.7359	5.4718	0.5360
	T11	0.0946	0.1847	0.2835	5.5344	0.6996
	T12	0.0922	0.1709	0.3299	4.4962	0.5582
	T13	0.0918	0.1764	0.4153	4.3478	0.6032
	T14	0.0828	0.1889	0.3112	4.3341	0.5991
	T15	0.0987	0.1998	0.3052	5.1290	0.5520
	T16	0.0843	0.2168	0.2493	4.9137	0.5620
