# Supplementary material for: Nutrient Diagnosis and Precise Fertilization Model Construction of ‘87-1’ Grape (Vitis vinifera L.) Cultivated in a Facility
Source: Plants (Basel). 2025 Oct 31;14(21):3345. doi: 10.3390/plants14213345 (PMC12611038; doi:10.3390/plants14213345)
Supplement: Supplementary file 1 [file plants-14-03345-s001.zip › Table S4.pdf]

Table S4. Annual results of orthogonal experiment on mineral element content ($mg\cdot g^{-1}$) in plant of high FQI subpopulations

Tissue	Year	Code	FBS					VS					MS				
			N	P	K	Ca	Mg	N	P	K	Ca	Mg	N	P	K	Ca	Mg
Fruit	2019	T7	26.93	12.82	44.13	17.34	13.74	10.44	6.91	27.35	10.44	2.34	5.80	5.53	22.10	5.13	3.16
	2020	T4	13.06	6.51	27.72	35.47	11.12	5.92	3.92	20.01	23.59	7.58	5.26	6.59	19.94	27.07	7.73
	2021	T9	22.85	5.20	32.59	14.93	3.70	8.57	2.05	18.97	7.21	1.73	13.16	4.60	30.72	13.46	2.18
	2022	T14	21.27	5.26	36.46	85.78	18.16	5.43	3.88	23.58	65.51	12.20	6.53	6.48	33.67	105.61	15.07
	2023	T11	27.84	7.07	33.87	17.30	3.89	8.81	2.73	19.52	9.33	1.88	11.31	4.78	30.42	12.26	2.28
	2019	T11	26.04	11.06	33.86	14.63	7.92	9.77	6.23	24.33	12.66	11.39	7.63	4.87	21.53	5.27	3.45
	2019	T15	29.23	12.25	36.92	15.43	8.32	9.55	5.37	26.74	10.26	5.82	7.49	7.12	18.34	12.25	6.05
	2023	T16	22.45	6.68	32.53	19.96	3.85	8.36	2.67	18.38	12.32	2.04	12.81	3.78	26.26	9.21	2.08
	2022	T5	18.83	5.17	36.23	88.88	18.77	5.75	4.72	26.32	76.48	14.31	5.58	3.10	22.52	64.97	11.69
	2022	T12	22.98	5.91	65.34	68.56	12.10	5.54	4.60	23.38	73.82	14.73	5.23	5.09	23.25	68.89	12.70
	2019	T12	24.99	12.40	34.86	11.57	3.90	9.54	5.23	21.13	7.00	2.62	6.51	5.37	20.50	7.40	2.63
	2019	T10	25.20	11.54	42.40	17.65	5.74	9.23	7.83	23.26	7.24	3.54	6.03	4.97	18.93	5.45	1.71
	2023	T9	22.26	5.14	28.37	16.02	2.79	8.75	3.07	19.54	7.69	1.51	11.33	3.48	28.63	9.33	2.25
	**Mean**		23.38	8.23	37.33	32.58	8.77	8.13	4.56	22.50	24.89	6.28	8.05	5.06	24.37	26.64	5.61
	**SD**		4.22	3.20	9.60	28.57	5.57	1.80	1.74	3.12	27.26	5.13	2.98	1.20	5.03	32.17	4.68
Leaf	2019	T7	33.85	9.72	16.81	22.04	6.12	38.70	9.74	14.59	28.60	4.93	29.74	12.04	19.89	32.86	6.31
	2020	T4	19.33	5.74	9.43	32.04	8.91	19.34	8.70	13.58	41.20	8.89	2.75	8.48	15.23	72.21	18.43
	2021	T9	28.83	4.91	21.01	25.52	3.30	30.90	4.87	15.32	29.26	3.08	26.97	5.08	14.50	49.97	4.22
	2022	T14	26.41	3.94	22.34	94.55	17.92	27.08	5.78	22.23	91.68	14.42	23.46	7.66	22.15	115.82	13.02
	2023	T11	33.33	5.86	13.12	24.40	4.35	26.84	4.92	12.90	32.29	4.48	25.93	5.62	13.68	43.35	5.59
	2019	T11	35.24	12.10	17.50	28.37	13.51	34.93	13.65	20.17	34.97	11.49	28.95	13.98	19.68	43.10	10.45
	2019	T15	34.90	11.37	44.50	28.80	6.95	34.85	12.42	18.21	33.47	5.97	30.71	13.67	20.59	41.18	8.20
	2023	T16	29.30	6.05	13.35	23.19	3.95	30.22	5.05	12.93	31.92	4.05	27.76	5.63	14.44	34.89	3.80
	2022	T5	26.98	3.94	20.15	96.57	19.46	23.54	5.94	19.25	93.35	14.45	26.76	5.79	19.87	124.56	18.31
	2022	T12	27.37	5.04	20.72	98.31	20.01	27.61	8.90	28.61	107.96	16.39	19.29	5.41	16.34	98.38	11.48
	2019	T12	34.58	10.62	17.18	22.53	5.72	33.13	13.85	18.11	24.52	4.85	29.58	14.06	21.83	38.82	18.48
	2019	T10	34.91	11.39	19.61	23.61	7.06	31.86	12.41	20.98	23.80	3.99	28.34	12.37	24.16	43.16	17.27
	2023	T9	29.01	3.94	12.65	20.95	3.52	26.53	4.76	13.40	30.39	3.46	30.13	4.34	16.06	42.76	4.79

	**Mean**		30.31	7.28	19.11	41.60	9.29	29.66	8.54	17.71	46.41	7.72	25.41	8.78	18.34	60.08	10.80
	**SD**		4.71	3.21	8.56	31.44	6.23	5.22	3.57	4.62	29.77	4.81	7.49	3.84	3.45	32.04	5.80
	2019	T7	9.79	9.89	42.85	25.85	8.81	9.53	8.31	48.35	17.48	3.61	6.67	7.91	52.14	21.29	5.34
	2020	T4	12.52	5.43	23.38	53.35	15.75	11.74	5.88	27.29	36.15	10.70	10.81	4.31	31.47	37.73	10.82
	2021	T9	9.15	4.48	21.99	17.67	3.32	7.08	3.12	31.90	27.31	5.08	8.53	4.01	30.97	37.77	6.76
	2022	T14	8.08	3.89	36.67	125.28	15.77	5.69	6.00	82.06	175.61	27.04	6.06	3.13	46.19	101.21	15.94
	2023	T11	12.33	6.32	20.20	18.80	4.38	7.03	4.83	23.94	24.48	6.98	9.95	5.21	23.16	41.61	9.82
	2019	T11	9.01	10.35	30.23	26.05	7.43	9.48	7.75	30.29	19.43	5.10	6.54	7.67	28.77	26.37	7.93
	2019	T15	8.97	9.34	25.31	16.74	2.40	11.82	6.59	31.52	17.96	3.91	7.27	7.06	29.70	26.66	6.35
**Petiole**	2023	T16	10.53	4.62	16.90	17.08	3.36	5.88	4.91	19.48	22.38	6.31	11.27	6.13	23.84	29.69	8.40
	2022	T5	8.29	3.22	28.43	144.17	22.39	5.89	2.77	46.19	159.28	23.79	6.16	1.49	28.93	113.76	21.23
	2022	T12	9.69	5.78	39.64	141.15	21.11	6.41	5.53	58.17	94.55	15.12	7.31	6.91	45.78	115.99	18.33
	2019	T12	9.92	10.74	36.85	22.89	7.30	11.04	7.12	33.23	17.96	4.68	6.24	9.46	35.84	23.72	6.99
	2019	T10	9.43	9.38	37.17	22.08	5.98	8.66	6.57	38.47	16.89	2.92	6.36	6.98	44.49	24.58	5.17
	2023	T9	7.19	3.21	17.25	20.26	3.50	7.40	2.77	25.26	25.88	5.60	9.18	3.24	26.07	34.19	7.53
	**Mean**		9.61	6.67	28.99	50.10	9.35	8.28	5.55	38.16	50.41	9.30	7.87	5.65	34.41	48.81	10.05
	**SD**		1.52	2.86	8.90	50.51	7.01	2.25	1.82	17.08	55.94	7.89	1.86	2.30	9.57	35.73	5.18
