# Supplementary material for: Nutrient Diagnosis and Precise Fertilization Model Construction of ‘87-1’ Grape (Vitis vinifera L.) Cultivated in a Facility
Source: Plants (Basel). 2025 Oct 31;14(21):3345. doi: 10.3390/plants14213345 (PMC12611038; doi:10.3390/plants14213345)
Supplement: Supplementary file 1 [file plants-14-03345-s001.zip › Table S5.pdf]

Table S5. Annual results of orthogonal experiment on mineral element content ($mg \cdot g^{-1}$) in soil of high FQI subpopulations

Stage	Year	Treatment	N	P	K	Ca	Mg
	2019	T7	0.0261	0.1287	0.2056	3.5301	0.6526
	2020	T4	0.0462	1.3939	0.4125	7.4192	1.1643
	2021	T9	0.1470	0.8287	0.8138	6.6696	0.9434
	2022	T14	0.3363	0.1712	1.3070	4.5946	0.4779
	2023	T11	0.2099	0.7215	0.2258	5.8392	0.7018
	2019	T11	0.0311	0.0397	0.1259	8.6300	0.7775
	2019	T15	0.0227	0.3360	0.2132	9.2077	0.5665
**GS**	2023	T16	0.1899	1.2891	0.5032	4.4336	0.9505
	2022	T5	0.3319	0.4975	1.7456	6.2490	0.5809
	2022	T12	0.2209	0.3224	1.4458	4.1268	0.4245
	2019	T12	0.0294	0.1002	0.2828	7.4371	0.7357
	2019	T10	0.0166	0.1100	0.4857	6.9691	0.4141
	2023	T9	0.1471	0.7871	0.4059	5.5610	0.7234
	**Mean**		0.1350	0.5174	0.6287	6.2052	0.7010
	**SD**		0.1169	0.4551	0.5345	1.7427	0.2211
	2019	T7	0.0233	0.4302	0.3951	3.5466	0.5817
	2020	T4	0.1221	2.1108	0.8285	7.4178	1.2079
	2021	T9	0.1171	0.7322	0.2535	7.0669	1.0522
	2022	T14	0.3948	0.5123	0.7985	4.5646	0.4565
	2023	T11	0.5899	0.9377	0.4478	5.4136	1.0974
	2019	T11	0.0218	0.3456	0.1344	3.5366	0.5440
	2019	T15	0.0277	0.3574	0.2261	3.7528	0.6922
**IFS**	2023	T16	0.4558	0.2489	0.4630	4.8377	0.6280
	2022	T5	0.2375	0.5015	1.4714	6.1204	0.4133
	2022	T12	0.0918	0.5248	1.5921	4.8312	0.4062
	2019	T12	0.0199	0.3763	0.2402	3.1477	0.4973
	2019	T10	0.0121	0.3665	0.4171	2.7017	0.3023
	2023	T9	0.4224	0.8449	0.8058	5.0913	0.6241
	**Mean**		0.1951	0.6376	0.6210	4.7715	0.6541
	**SD**		0.2023	0.4881	0.4651	1.4617	0.2871
	2019	T7	0.0182	0.4597	0.6733	3.2290	0.4558
	2020	T4	0.0575	1.5905	0.7878	8.2306	1.2662
	2021	T9	1.1602	1.0739	1.2103	6.5841	0.6929
	2022	T14	0.7895	0.1122	0.7257	4.8787	0.5868
	2023	T11	0.4185	0.3211	0.2671	5.5759	0.9118
	2019	T11	0.0324	0.3359	0.1476	3.9456	0.8095
	2019	T15	0.0190	0.2903	0.3840	3.0551	0.4738
**EBS**	2023	T16	0.3377	0.3342	0.2768	4.9260	0.5363
	2022	T5	0.2630	0.5472	0.6181	7.5856	0.6702
	2022	T12	0.3707	0.1379	0.9469	4.1237	0.5240
	2019	T12	0.0253	0.3901	0.4401	2.6862	0.6316
	2019	T10	0.0171	0.2853	0.4866	3.2745	0.4280
	2023	T9	0.3001	0.2563	0.4173	4.6168	0.6021
	**Mean**		0.2930	0.4719	0.5678	4.8240	0.6607

	**SD**		0.3470	0.4125	0.2992	1.7526	0.2288
**VS**	2019	T7	0.0270	0.2811	0.7855	3.5024	0.6761
	2020	T4	0.0932	2.8284	1.0753	7.0221	0.9326
	2021	T9	0.3547	1.0437	0.6487	7.0953	0.7156
	2022	T14	0.7351	0.2934	0.7310	3.6015	0.5253
	2023	T11	0.4136	0.1756	0.2544	5.6199	0.8418
	2019	T11	0.0306	0.1242	0.2620	3.0432	0.7647
	2019	T15	0.0249	0.2133	0.4816	3.8842	0.6214
	2023	T16	0.3298	0.1609	0.3114	5.9508	1.1971
	2022	T5	0.1533	0.1185	1.2120	6.0536	0.4767
	2022	T12	0.5465	0.4663	0.9968	3.5709	0.5309
	2019	T12	0.0318	0.1980	0.4015	3.0755	0.7938
	2019	T10	0.0236	0.1900	0.4843	3.9529	0.5907
	2023	T9	0.1245	0.1786	0.5089	6.1774	1.1366
	**Mean**		0.2222	0.4825	0.6272	4.8115	0.7541
	**SD**		0.2335	0.7464	0.3155	1.5253	0.2263
**MS**	2019	T7	0.0307	0.2197	0.8122	2.8150	0.7681
	2020	T4	0.0653	1.1178	0.4778	8.7768	1.1864
	2021	T9	0.1179	1.2576	0.7665	6.9011	0.5016
	2022	T14	0.3081	0.2494	0.8994	3.2988	0.3911
	2023	T11	0.0946	0.1847	0.2835	5.5344	0.6996
	2019	T11	0.0271	0.1550	0.2652	2.7335	0.6776
	2019	T15	0.0269	0.1960	0.3629	3.2846	0.6723
	2023	T16	0.0843	0.2168	0.2493	4.9137	0.5620
	2022	T5	0.2469	0.1775	0.9081	5.7855	0.5571
	2022	T12	0.6907	0.3344	1.1135	3.6140	0.5057
	2019	T12	0.0301	0.2424	0.4428	2.6589	0.7528
	2019	T10	0.0207	0.1801	0.6124	2.6753	0.5176
	2023	T9	0.1075	0.1707	0.3605	5.6561	0.5916
	**Mean**		0.1424	0.3617	0.5811	4.5114	0.6449
	**SD**		0.1869	0.3706	0.2894	1.9294	0.1964
