# Supplementary material for: Nutrient Diagnosis and Precise Fertilization Model Construction of ‘87-1’ Grape (Vitis vinifera L.) Cultivated in a Facility
Source: Plants (Basel). 2025 Oct 31;14(21):3345. doi: 10.3390/plants14213345 (PMC12611038; doi:10.3390/plants14213345)
Supplement: Supplementary file 1 [file plants-14-03345-s001.zip › Table S6.pdf]

**Table S6. Correlation coefficients between the mineral element contents ($mg \cdot g^{-1}$) and quality characteristics of '87-1' grape**

| Type | Stage | Quality | N | P | K | Ca | Mg |
|---|---|---|---|---|---|---|---|
| **Soil** | GS | SFW | 0.192 | 0.385 | -0.026 | 0.001 | 0.378 |
| | | TSS | -0.009 | -0.669* | 0.086 | -0.164 | -0.513 |
| | | FF | 0.262 | 0.601* | 0.144 | 0.042 | 0.508 |
| | | FQI | 0.130 | 0.018 | -0.033 | -0.076 | 0.105 |
| | IFS | SFW | 0.398 | 0.102 | -0.094 | 0.384 | 0.214 |
| | | TSS | -0.311 | -0.771** | -0.167 | -0.541 | -0.477 |
| | | FF | 0.325 | 0.424 | -0.033 | 0.787** | 0.656* |
| | | FQI | 0.144 | -0.304 | -0.413 | 0.175 | 0.284 |
| | EBS | SFW | 0.252 | 0.231 | -0.138 | 0.484 | 0.185 |
| | | TSS | 0.188 | -0.603* | 0.089 | -0.672* | -0.655* |
| | | FF | 0.670* | 0.610* | 0.452 | 0.727** | 0.385 |
| | | FQI | 0.734** | 0.006 | 0.296 | 0.015 | -0.212 |
| | VS | SFW | -0.167 | -0.036 | -0.091 | 0.633* | 0.397 |
| | | TSS | 0.250 | -0.603* | -0.274 | -0.689** | -0.413 |
| | | FF | 0.158 | 0.389 | 0.140 | 0.870** | 0.185 |
| | | FQI | 0.280 | -0.233 | -0.341 | 0.224 | -0.020 |
| | MS | SFW | -0.396 | 0.114 | -0.278 | 0.456 | -0.093 |
| | | TSS | 0.333 | -0.300 | 0.310 | -0.740** | -0.591* |
| | | FF | -0.162 | 0.695** | -0.002 | 0.784** | 0.004 |
| | | FQI | -0.108 | 0.305 | 0.030 | 0.054 | -0.467 |
| **Fruit** | IFS | SFW | -0.218 | -0.384 | -0.628* | -0.129 | -0.177 |
| | | TSS | 0.669* | 0.328 | 0.606* | -0.020 | 0.001 |
| | | FF | -0.350 | -0.581* | -0.486 | -0.058 | -0.217 |
| | | FQI | 0.399 | -0.143 | -0.106 | -0.281 | -0.351 |
| | VS | SFW | 0.125 | -0.314 | -0.285 | -0.218 | -0.253 |
| | | TSS | 0.273 | 0.229 | 0.330 | 0.090 | 0.136 |
| | | FF | -0.140 | -0.652* | -0.518 | -0.139 | -0.309 |
| | | FQI | 0.324 | -0.413 | -0.256 | -0.289 | -0.394 |
| | MS | SFW | 0.549 | -0.681* | 0.341 | -0.252 | -0.361 |
| | | TSS | -0.006 | 0.087 | 0.075 | 0.090 | 0.053 |
| | | FF | 0.590* | -0.309 | 0.477 | -0.083 | -0.193 |
| | | FQI | 0.702** | -0.204 | 0.555* | -0.193 | -0.351 |
| **Leaf** | IFS | SFW | -0.104 | -0.311 | -0.394 | -0.252 | -0.278 |
| | | TSS | 0.575* | 0.322 | 0.427 | 0.172 | 0.193 |
| | | FF | -0.388 | -0.535 | -0.210 | -0.147 | -0.340 |
| | | FQI | 0.287 | -0.111 | 0.145 | -0.223 | -0.376 |
| | VS | SFW | -0.195 | -0.522 | -0.575* | -0.272 | -0.300 |
| | | TSS | 0.717** | 0.305 | 0.557* | 0.166 | 0.160 |
| | | FF | -0.413 | -0.657* | -0.508 | -0.163 | -0.328 |
| | | FQI | 0.357 | -0.359 | -0.215 | -0.247 | -0.391 |
| | MS | SFW | 0.196 | -0.403 | -0.307 | -0.101 | -0.309 |
| | | TSS | 0.565* | 0.265 | 0.270 | -0.084 | -0.278 |
| | | FF | -0.168 | -0.591* | -0.553* | 0.040 | -0.258 |
| | | FQI | 0.455 | -0.249 | -0.303 | -0.263 | -0.639* |

| | | | | | | | |
|---|---|---|---|---|---|---|---|
| | | SFW | -0.222 | -0.452 | -0.599* | -0.233 | -0.248 |
| | IFS | TSS | -0.345 | 0.398 | 0.523 | 0.072 | -0.090 |
| | | FF | 0.120 | -0.581* | -0.612* | -0.142 | -0.167 |
| | | FQI | -0.119 | -0.111 | -0.235 | -0.316 | -0.483 |
| | | SFW | -0.357 | -0.580* | -0.398 | -0.053 | -0.054 |
| **Petiole** | VS | TSS | -0.164 | 0.358 | 0.383 | 0.022 | -0.080 |
| | | FF | -0.275 | -0.721** | -0.305 | -0.009 | 0.014 |
| | | FQI | -0.346 | -0.289 | -0.105 | -0.168 | -0.229 |
| | | SFW | 0.299 | -0.586* | -0.526 | -0.171 | -0.097 |
| | MS | TSS | -0.546 | 0.527 | 0.419 | 0.116 | -0.049 |
| | | FF | 0.455 | -0.638* | -0.447 | -0.028 | -0.014 |
| | | FQI | 0.058 | -0.075 | -0.154 | -0.161 | -0.279 |

** Significant at 0.01 probability level

* Significant at 0.05 probability level
