# Supplementary material for: Nutrient Diagnosis and Precise Fertilization Model Construction of ‘87-1’ Grape (Vitis vinifera L.) Cultivated in a Facility
Source: Plants (Basel). 2025 Oct 31;14(21):3345. doi: 10.3390/plants14213345 (PMC12611038; doi:10.3390/plants14213345)
Supplement: Supplementary file 1 [file plants-14-03345-s001.zip › Table S7.pdf]

Table S7. The relationship between the cumulative variance of plant and soil elements and various fruit quality

Quality	Type	Stage_(Tissue)_Element	Functional relationship	R^2	A	B	Y=-B/3A	Number and ratio of high subpopulation	Optimum range ($mg \cdot g^{-1}$)
SFW	Plant	FBS_L_N	y = 253.52x3 - 383.59x2 + 33.104x + 98.92	0.9950	253.520	-383.590	0.5044	41 and 51.25%	7.036 - 12.516
		VS_P_P	y = 353.9x3 - 544.57x2 + 99.905x + 92.096	0.9785	353.900	-544.570	0.5129		0.113 - 8.399
		FBS_F_K	y = 2.2519x3 + 36.442x2 - 117.02x + 77.977	0.9660	2.252	36.442	-5.3943		16.407 - 30.708
		FBS_F_Ca	y = 228.4x3 - 376.34x2 + 53.763x + 97.17	0.9937	228.400	-376.340	0.5492		21.777 - 114.665
		FBS_F_Mg	y = 151.6x3 - 237.24x2 - 6.5379x + 97.378	0.9896	151.600	-237.240	0.5216		4.775 - 31.028
		R	y = 328.09x3 - 507.58x2 + 86.847x + 95.607	0.9903	328.090	-507.580	0.5157		
	Soil	GS_N	y = 49.574x3 - 57.244x2 - 79.594x + 89.833	0.9881	49.574	-57.244	0.3849	27 and 33.75%	0.017 - 0.589
		GS_P	y = 94.545x3 - 152.97x2 - 38.555x + 96.939	0.9931	94.545	-152.970	0.5393		0.040 - 1.387
		GS_K	y = 97.247x3 - 167.36x2 - 24.895x + 99.867	0.9950	97.247	-167.360	0.5737		0.126 - 1.746
		GS_Ca	y = 128.3x3 - 245.75x2 + 24.921x + 94.038	0.9892	128.300	-245.750	0.6385		3.965 - 9.250
		GS_Mg	y = 198.35x3 - 348.34x2 + 56.513x + 97.994	0.9949	198.350	-348.340	0.5854		0.318 - 2.382
		GS_R	y = 91.592x3 - 132.76x2 - 51.998x + 95.41	0.9928	91.592	-132.760	0.4832		
		IFS_N	y = 218.29x3 - 339.23x2 + 29.37x + 94.86	0.9923	218.290	-339.230	0.5180	29 and 36.25%	0.012 - 1.178
		IFS_P	y = 272.67x3 - 407.57x2 + 38.764x + 99.005	0.9948	272.670	-407.570	0.4982		0.194 - 1.795
		IFS_K	y = 98.87x3 - 187.58x2 - 4.8993x + 98.588	0.9934	98.870	-187.580	0.6324		0.134 - 1.471
		IFS_Ca	y = 110.76x3 - 181.44x2 - 22.453x + 96.779	0.9952	110.760	-181.440	0.5460		2.702 - 7.990
		IFS_Mg	y = 138.07x3 - 252.56x2 + 20.603x + 99.787	0.9945	138.070	-252.560	0.6097		0.299 - 1.379
		IFS_R	y = 125.55x3 - 209.72x2 - 4.8367x + 95.133	0.9910	125.550	-209.720	0.5568		
		EBS_N	y = -16.778x3 + 69.745x2 - 138.62x + 87.247	0.9824	-16.778	69.745	1.3856	41 and 51.25%	0.017 - 1.160
		EBS_P	y = -26.642x3 + 91.954x2 - 138.13x + 73.689	0.9506	-26.642	91.954	1.1505		0.205 - 1.701
		EBS_K	y = 86.826x3 - 66.978x2 - 120.34x + 102.3	0.9940	86.826	-66.978	0.2571		0.148 - 1.253
		EBS_Ca	y = -22.682x3 + 85.121x2 - 158.18x + 97.363	0.9879	-22.682	85.121	1.2509		3.229 - 9.690
		EBS_Mg	y = 209.86x3 - 343.55x2 + 39.287x + 98.615	0.9951	209.860	-343.550	0.5457		0.428 - 1.708
		EBS_R	y = 40.886x3 - 63.374x2 - 49.73x + 78.256	0.9688	40.886	-63.374	0.5167		
		VS_N	y = 104.74x3 - 119.97x2 - 82.701x + 103.48	0.9938	104.740	-119.970	0.3818		0.024 - 0.480
		VS_P	y = 195.13x3 - 329.53x2 + 41.885x + 96.781	0.9963	195.130	-329.530	0.5629		0.119 - 4.143
		VS_K	y = 157.98x3 - 292.92x2 + 44.038x + 96.318	0.9927	157.980	-292.920	0.6181		0.168 - 1.252

		VS_Ca	y = -3.5707x3 + 26.879x2 - 116.47x + 99.112	0.9912	-3.571	26.879	2.5092		3.043 - 7.609
		VS_Mg	y = 122.7x3 - 232.76x2 + 21.363x + 98.732	0.9945	122.700	-232.760	0.6323		0.303 - 1.216
		VS_R	y = -102.43x3 + 206.45x2 - 160.47x + 57.516	0.7741	-102.430	206.450	0.6718	25 and 31.25%	
		MS_N	-252.06x3 + 481.13x2 - 298.19x + 67.823	0.8369	-252.060	481.130	0.6363	27 and 33.75%	0.021 - 0.539
		MS_P	162.06x3 - 238.34x2 - 17.564x + 98.865	0.9948	162.060	-238.340	0.4902		0.119 - 1.214
		MS_K	204.19x3 - 322.79x2 + 22.161x + 101.38	0.9955	204.190	-322.790	0.5269		0.152 - 1.209
		MS_Ca	-44.558x3 + 117.89x2 - 156.81x + 83.73	0.9705	-44.558	117.890	0.8819		2.675 - 7.902
		MS_Mg	82.661x3 - 128.15x2 - 43.731x + 102.91	0.9921	82.661	-128.150	0.5168		0.321 - 1.156
		MS_R	146.84x3 - 268.98x2 + 36.569x + 95.828	0.9926	146.840	-268.980	0.6106		
		FBS_L_N	y = 182.47x3 - 272.63x2 - 2.7252x + 101.49	0.9902	182.470	-272.630	0.4980		7.333 - 11.927
		VS_P_P	y = -27.1x3 - 44.322x2 - 9.6701x + 100.14	0.9803	-27.100	-44.322	-0.5452		0.113 - 6.483
		FBS_F_K	y = 192.27x3 - 339.03x2 + 60.847x + 98.69	0.9912	192.270	-339.030	0.5878		16.617 - 27.920
	Plant	FBS_F_Ca	y = 177.65x3 - 245.25x2 - 27.761x + 101.73	0.9912	177.650	-245.250	0.4602		21.777 - 107.960
		FBS_F_Mg	y = 181.9x3 - 281.06x2 + 8.7735x + 100.2	0.9931	181.900	-281.060	0.5150		6.444 - 20.982
		R	y = 105.84x3 - 207.04x2 + 11.232x + 99.738	0.9933	105.840	-207.040	0.6521	23 and 28.75%	
		GS_N	y = 57.478x3 - 81.09x2 - 45.921x + 73.03	0.9571	57.478	-81.090	0.4703		0.020 - 0.841
		GS_P	y = 283.97x3 - 435.47x2 + 65.955x + 89.325	0.9906	283.970	-435.470	0.5112	36 and 45.00%	0.040 - 1.387
		GS_K	y = 206.21x3 - 272.46x2 - 26.865x + 97.1	0.9910	206.210	-272.460	0.4404		0.126 - 1.539
		GS_Ca	y = 193.85x3 - 273.79x2 - 7.5609x + 96.337	0.9895	193.850	-273.790	0.4708		3.530 - 9.250
		GS_Mg	y = 160.75x3 - 217.1x2 - 29.266x + 90.468	0.9895	160.750	-217.100	0.4502		0.366 - 2.382
		GS_R	y = -5.8261x3 + 66.201x2 - 131.66x + 73.749	0.9177	-5.826	66.201	3.7876		
		IFS_N	y = 154.04x3 - 234.46x2 - 9.1185x + 95.576	0.9945	154.040	-234.460	0.5074		0.014 - 1.034
		IFS_P	y = 237.01x3 - 373.35x2 + 37.101x + 98.888	0.9947	237.010	-373.350	0.5251		0.221 - 1.795
		IFS_K	y = 182.9x3 - 300.24x2 + 26.765x + 95.057	0.9961	182.900	-300.240	0.5472		0.134 - 1.696
		IFS_Ca	y = 182.45x3 - 307.97x2 + 34.652x + 100.74	0.9936	182.450	-307.970	0.5627	34 and 42.50%	2.160 - 9.749
		IFS_Mg	y = 272.38x3 - 414.03x2 + 48.244x + 98.471	0.9953	272.380	-414.030	0.5067		0.308 - 1.886
		IFS_R	y = 135.08x3 - 191.99x2 - 20.405x + 87.819	0.9774	135.080	-191.990	0.4738		
**TSS**		EBS_N	y = 242.46x3 - 362.03x2 + 31.921x + 94.634	0.9935	242.460	-362.030	0.4977		0.018 - 1.160
		EBS_P	y = 244.26x3 - 365.54x2 + 25.696x + 98.123	0.9945	244.260	-365.540	0.4988		0.103 - 1.701
	Soil	EBS_K	y = 341.91x3 - 530.58x2 + 96.438x + 96.396	0.9940	341.910	-530.580	0.5173	36 and 45.00%	0.148 - 1.561
		EBS_Ca	y = 325.84x3 - 491.79x2 + 71.299x + 99.585	0.9932	325.840	-491.790	0.5031		2.686 - 9.690
		EBS_Mg	y = 76.927x3 - 96.451x2 - 44.117x + 66.632	0.9307	76.927	-96.451	0.4179		0.335 - 1.708

		EBS_R	y = 134.12x3 - 195.73x2 - 18.432x + 88.969	0.9876	134.120	-195.730	0.4865		
		VS_N	y = 296.03x3 - 436.22x2 + 49.247x + 94.778	0.9954	296.030	-436.220	0.4912		0.022 - 0.966
		VS_P	y = 262.29x3 - 408.52x2 + 57.932x + 91.668	0.9953	262.290	-408.520	0.5192		0.124 - 4.143
		VS_K	y = 249.97x3 - 363.77x2 + 17.855x + 99.707	0.9956	249.970	-363.770	0.4851		0.236 - 2.276
		VS_Ca	y = 257.08x3 - 406.7x2 + 53.288x + 99.74	0.9960	257.080	-406.700	0.5273	35 and 43.75%	3.043 - 8.187
		VS_Mg	y = 169.15x3 - 259.71x2 - 0.8592x + 96.356	0.9952	169.150	-259.710	0.5118		0.313 - 1.226
		VS_R	y = 40.621x3 - 23.376x2 - 71.405x + 56.523	0.8424	40.621	-23.376	0.1918		
		MS_N	y = 120.64x3 - 146.81x2 - 64.921x + 96.025	0.9925	120.640	-146.810	0.4056		0.027 - 0.691
		MS_P	y = 261.11x3 - 422.83x2 + 69.505x + 98.278	0.9957	261.110	-422.830	0.5398	34 and 42.50%	0.135 - 1.258
		MS_K	y = 329.88x3 - 484.41x2 + 59.061x + 98.569	0.9946	329.880	-484.410	0.4895		0.234 - 1.448
		MS_Ca	y = 200.01x3 - 284.73x2 - 10.23x + 98.598	0.9962	200.010	-284.730	0.4745		2.659 - 8.566
		MS_Mg	y = 182.81x3 - 269.13x2 - 0.3507x + 93.64	0.9937	182.810	-269.130	0.4907		0.225 - 1.480
		MS_R	y = 144.51x3 - 209.85x2 - 13.444x + 87.806	0.9868	144.510	-209.850	0.4840		
		FBS_L_N	y = 14.704x3 + 62.241x2 - 172x + 98.175	0.9964	14.704	62.241	-1.4110		7.036 - 12.516
		VS_P_P	y = -218.38x3 + 424.5x2 - 301.37x + 94.307	0.9691	-218.380	424.500	0.6480	22 and 27.50%	3.097 - 7.122
	Plant	FBS_F_K	y = 69.931x3 - 116.81x2 - 39.146x + 98.979	0.9959	69.931	-116.810	0.5568		15.043 - 30.708
		FBS_F_Ca	y = 184.56x3 - 205.68x2 - 76.186x + 103.25	0.9880	184.560	-205.680	0.3715		23.801 - 93.348
		FBS_F_Mg	y = 134.75x3 - 154.74x2 - 76.911x + 102.8	0.9928	134.750	-154.740	0.3828		4.775 - 28.703
		R	y = -9.4316x3 + 80.483x2 - 165.29x + 97.782	0.9961	-9.432	80.483	2.8444		
		GS_N	y = 59.058x3 - 63.729x2 - 74.119x + 81.327	0.9877	59.058	-63.729	0.3597		0.017 - 0.417
		GS_P	y = 124.03x3 - 137.12x2 - 52.383x + 69.334	0.9649	124.030	-137.120	0.3685		0.036 - 1.542
		GS_K	y = 97.328x3 - 60.911x2 - 132.79x + 101.26	0.9894	97.328	-60.911	0.2086		0.126 - 1.955
		GS_Ca	y = 226.13x3 - 303.82x2 - 10.744x + 90.233	0.9947	226.130	-303.820	0.4479	38 and 47.50%	3.252 - 9.208
		GS_Mg	y = 169.95x3 - 205.03x2 - 57.529x + 97.226	0.9949	169.950	-205.030	0.4021		0.209 - 1.688
		GS_R	y = 89.992x3 - 93.4x2 - 56.849x + 62.057	0.9408	89.992	-93.400	0.3460		
		IFS_N	y = 179.05x3 - 223.59x2 - 52.547x + 100.02	0.9969	179.050	-223.590	0.4163		0.012 - 1.034
		IFS_P	y = -0.1377x3 + 61.544x2 - 157.51x + 100.4	0.9967	-0.138	61.544	148.9809		0.215 - 2.111
		IFS_K	y = 48.426x3 - 9.3615x2 - 131.25x + 96.88	0.9911	48.426	-9.362	0.0644		0.105 - 1.946
		IFS_Ca	y = 197.17x3 - 269.34x2 - 24.778x + 100.46	0.9959	197.170	-269.340	0.4553	37 and 46.25%	2.702 - 11.000
		IFS_Mg	y = 170.3x3 - 221.35x2 - 43.216x + 99.953	0.9930	170.300	-221.350	0.4333		0.241 - 1.391
**FF**		IFS_R	y = 162.14x3 - 203.57x2 - 43.037x + 88.354	0.9902	162.140	-203.570	0.4185		
		EBS_N	y = 182.88x3 - 234.04x2 - 41.773x + 97.535	0.9960	182.880	-234.040	0.4266		0.017 - 1.160

	EBS_P	y = 114.18x3 - 91.255x2 - 116.54x + 98.772	0.9941	114.180	-91.255	0.2664		0.103 - 1.591
Soil	EBS_K	y = 24.525x3 - 28.443x2 - 65.878x + 69.283	0.9626	24.525	-28.443	0.3866		0.122 - 1.561
	EBS_Ca	y = 69.541x3 - 90.941x2 - 71.602x + 100.89	0.9962	69.541	-90.941	0.4359	40 and 50.00%	2.686 - 8.354
	EBS_Mg	y = -16.868x3 + 50.435x2 - 117.06x + 87.884	0.9893	-16.868	50.435	0.9967		0.335 - 1.375
	EBS_R	y = 192.93x3 - 252.19x2 - 32.989x + 98.147	0.9958	192.930	-252.190	0.4357		
	VS_N	y = -4.2043x3 + 58.599x2 - 121.73x + 68.29	0.9581	104.740	-119.970	0.3818		0.018 - 0.414
	VS_P	y = -45.864x3 + 95.305x2 - 127.58x + 86.891	0.9891	195.130	-329.530	0.5629		0.119 - 2.828
	VS_K	y = -48.84x3 + 125.48x2 - 173.23x + 98.79	0.9954	157.980	-292.920	0.6181		0.158 - 1.212
	VS_Ca	y = 123.51x3 - 131.62x2 - 86.486x + 102.15	0.9939	-3.571	26.879	2.5092		3.043 - 9.500
	VS_Mg	y = -50.739x3 + 138.98x2 - 145.03x + 57.464	0.8475	122.700	-232.760	0.6323		0.335 - 1.197
	VS_R	y = 20.916x3 + 13.4x2 - 97.672x + 66.528	0.9492	-102.430	206.450	0.6718	19 and 23.75%	
	MS_N	y = 131.37x3 - 138.73x2 - 82.386x + 94.176	0.9931	131.370	-138.730	0.3520		0.021 - 0.247
	MS_P	y = -9.7691x3 + 22.957x2 - 106.5x + 99.558	0.9963	-9.769	22.957	0.7833	12 and 15.00%	0.078 - 1.258
	MS_K	y = 46.857x3 - 23.073x2 - 110.74x + 88.51	0.9918	46.857	-23.073	0.1641		0.127 - 0.908
	MS_Ca	y = 5.8324x3 + 52.727x2 - 131.76x + 73.799	0.9717	5.832	52.727	-3.0135		2.675 - 8.777
	MS_Mg	y = 181.94x3 - 232.11x2 - 44.291x + 99.338	0.9947	181.940	-232.110	0.4253		0.502 - 1.186
	MS_R	y = 136.42x3 - 152.08x2 - 82.849x + 101.78	0.9963	136.420	-152.080	0.3716		
