# Supplementary material for: Nutrient Diagnosis and Precise Fertilization Model Construction of ‘87-1’ Grape (Vitis vinifera L.) Cultivated in a Facility
Source: Plants (Basel). 2025 Oct 31;14(21):3345. doi: 10.3390/plants14213345 (PMC12611038; doi:10.3390/plants14213345)
Supplement: Supplementary file 1 [file plants-14-03345-s001.zip › Table S8.pdf]

Table S8. Dry weight (g) of various tissues at different growth stages

Year	Tissue	Stage						
		GS	IFS	EBS	SDS	VS	MS	DS
2019	Root	99.2	138.5	154.8	180.3	180.7	255.7	319.1
	Trunk	259.5	262.3	284.6	303.3	325.5	344.6	385.2
	Main stem	369.8	432.1	446.2	455.4	474.6	624.5	633.3
	Shoot	-	98.4	118.9	195.0	203.8	458.9	839.4
	Leaf	-	95.8	111.1	120.0	131.3	155.0	172.9
	Petiole	-	21.7	24.3	28.0	29.9	31.7	58.9
	Inflorescence/Fruit	-	7.7	15.4	290.0	451.7	612.1	612.1
	Total per plant	728.5	1056.5	1155.3	1571.9	1797.5	2482.6	3020.8
2020	Root	118.4	161.8	192.1	194.9	211.3	267.7	373.5
	Trunk	300.0	328.1	355.0	361.2	365.8	409.1	446.0
	Main stem	415.5	533.6	536.5	552.9	592.5	704.5	720.4
	Shoot	-	103.4	115.4	188.9	211.5	485.4	898.7
	Leaf	-	103.9	110.7	123.5	137.2	158.2	175.3
	Petiole	-	21.3	23.9	29.4	29.9	31.7	60.3
	Inflorescence/Fruit	-	7.6	16.2	286.0	426.0	610.6	610.6
	Total per plant	833.9	1259.6	1349.8	1736.9	1974.1	2667.2	3284.6
2021	Root	122.4	169.0	195.9	214.2	243.2	315.1	378.6
	Trunk	315.3	335.4	353.1	388.6	424.4	426.7	467.6
	Main stem	454.5	534.8	569.3	611.5	607.6	717.0	785.6
	Shoot	-	106.3	121.0	193.9	194.1	487.4	908.3
	Leaf	-	99.7	114.6	120.9	140.2	160.1	178.6
	Petiole	-	21.7	23.8	28.6	28.2	31.0	63.4
	Inflorescence/Fruit	-	7.6	14.9	290.8	447.4	654.5	654.5
	Total per plant	892.3	1274.5	1392.5	1848.5	2085.1	2792.0	3436.7
