# Supplementary material for: Nutrient Diagnosis and Precise Fertilization Model Construction of ‘87-1’ Grape (Vitis vinifera L.) Cultivated in a Facility
Source: Plants (Basel). 2025 Oct 31;14(21):3345. doi: 10.3390/plants14213345 (PMC12611038; doi:10.3390/plants14213345)
Supplement: Supplementary file 1 [file plants-14-03345-s001.zip › Table S9.pdf]

Table S9. Nutrient concentration (mg·g^{-1}) of various tissues at different growth stages

Year	Stage_Tissue	N	P	K	Ca	Mg
2019	GS_Root	15.912	6.308	4.880	15.176	2.655
	GS_Trunk	4.593	1.263	3.365	20.330	2.329
	GS_Main stem	5.456	1.954	6.107	12.065	2.057
	IFS_Root	13.604	5.312	3.594	14.628	2.490
	IFS_Trunk	2.914	1.546	3.781	16.003	2.223
	IFS_Main stem	3.301	1.783	6.558	12.903	1.908
	IFS_Shoot	8.085	2.764	13.425	15.466	3.791
	IFS_Leaf	30.265	4.399	10.924	15.726	3.064
	IFS_Petiole	8.830	3.775	17.303	14.051	3.182
	IFS_Inflorescence	22.087	4.346	23.117	10.388	2.604
	EBS_Root	18.590	5.904	3.749	15.308	2.736
	EBS_Trunk	3.802	1.946	5.053	14.227	2.845
	EBS_Main stem	4.430	1.995	5.017	12.651	1.799
	EBS_Shoot	8.578	2.588	15.252	13.487	4.610
	EBS_Leaf	34.536	3.690	10.902	18.032	2.990
	EBS_Petiole	8.804	3.455	21.871	15.864	3.168
	EBS_Fruit	18.741	4.624	22.247	8.028	2.249
	SDS_Root	15.018	7.340	3.208	13.719	2.676
	SDS_Trunk	2.930	1.910	3.856	13.067	2.513
	SDS_Main stem	3.348	1.475	5.258	9.706	1.812
	SDS_Shoot	5.200	2.261	11.168	11.446	3.639
	SDS_Leaf	26.181	4.352	12.237	17.583	2.646
	SDS_Petiole	8.066	3.085	18.998	15.212	3.674
	SDS_Fruit	15.994	2.814	15.800	6.918	1.227
	VS_Root	15.127	8.395	3.916	15.855	2.512
	VS_Trunk	11.520	1.896	6.530	16.489	2.476
	VS_Main stem	3.764	1.845	5.349	6.848	1.317
	VS_Shoot	5.552	2.005	10.926	4.888	1.241
	VS_Leaf	18.297	2.833	8.861	18.470	2.931
	VS_Petiole	6.632	2.986	19.801	17.719	4.506
	VS_Fruit	6.576	2.077	16.588	7.060	2.439
	MS_Root	14.365	8.076	3.364	8.172	1.576
	MS_Trunk	7.704	1.246	3.579	19.210	2.426
	MS_Main stem	2.978	1.677	4.521	14.745	1.261
	MS_Shoot	3.885	1.852	7.360	5.337	1.126
	MS_Leaf	18.977	3.322	8.576	31.718	2.826
	MS_Petiole	6.162	2.829	16.060	19.551	6.126
	MS_Fruit	5.779	1.763	12.049	6.677	1.039
	DS_Root	13.253	6.432	3.136	9.090	1.448
	DS_Trunk	4.170	2.153	3.412	15.312	1.707
	DS_Main stem	4.419	2.036	4.260	9.340	1.695
	DS_Shoot	5.803	2.083	6.417	8.899	1.589
	DS_Leaf	8.273	2.498	6.451	38.183	3.404
	DS_Petiole	5.552	2.152	12.445	21.809	7.943
	GS_Root	17.008	5.689	4.454	13.671	2.782

	GS_Trunk	4.452	1.329	3.522	18.726	2.529
	GS_Main stem	5.535	1.919	5.742	12.559	2.094
	IFS_Root	13.716	5.116	3.546	13.569	2.240
	IFS_Trunk	2.979	1.424	3.966	15.394	2.171
	IFS_Main stem	2.900	1.673	5.714	13.797	1.836
	IFS_Shoot	7.674	2.638	13.002	14.548	4.188
	IFS_Leaf	30.391	4.247	10.510	17.902	3.233
	IFS_Petiole	9.196	3.469	16.897	15.269	3.227
	IFS_Inflorescence	21.226	4.167	24.449	10.663	2.709
	EBS_Root	16.906	5.888	3.404	13.546	3.057
	EBS_Trunk	3.950	1.799	5.293	14.697	3.166
	EBS_Main stem	3.923	1.767	5.251	12.912	1.815
	EBS_Shoot	8.365	2.619	14.402	13.180	4.745
	EBS_Leaf	33.026	4.141	10.448	18.822	2.814
	EBS_Petiole	8.489	3.357	22.216	14.824	3.254
	EBS_Fruit	18.773	4.455	19.734	6.951	2.007
	SDS_Root	14.991	6.718	3.552	13.733	2.528
	SDS_Trunk	3.058	1.824	4.069	12.753	2.459
	SDS_Main stem	3.281	1.425	5.105	9.538	1.823
	SDS_Shoot	5.212	2.276	11.104	11.689	3.601
2020	SDS_Leaf	28.971	4.389	11.993	17.209	2.742
	SDS_Petiole	7.321	3.164	20.158	14.786	3.628
	SDS_Fruit	15.418	3.154	16.275	6.436	1.256
	VS_Root	15.806	8.383	4.236	13.937	2.722
	VS_Trunk	11.587	1.809	6.077	16.275	2.342
	VS_Main stem	3.911	1.844	5.642	7.844	1.293
	VS_Shoot	5.775	2.061	9.374	4.933	1.296
	VS_Leaf	17.643	3.015	8.866	17.638	2.629
	VS_Petiole	7.019	2.936	21.094	16.989	4.436
	VS_Fruit	7.530	2.082	14.587	7.884	2.399
	MS_Root	15.392	8.059	3.737	8.927	1.696
	MS_Trunk	7.324	1.396	3.223	19.289	2.228
	MS_Main stem	2.909	1.707	4.303	13.736	1.353
	MS_Shoot	3.971	1.903	7.255	5.368	1.219
	MS_Leaf	20.241	3.368	9.254	30.206	2.782
	MS_Petiole	6.551	2.843	17.368	22.220	6.529
	MS_Fruit	6.395	1.747	12.653	6.374	1.049
	DS_Root	14.415	5.782	3.499	9.000	1.490
	DS_Trunk	4.109	1.946	3.586	17.709	1.822
	DS_Main stem	4.548	1.867	4.749	8.860	1.520
	DS_Shoot	5.164	2.246	6.607	9.977	1.502
	DS_Leaf	8.654	2.537	7.108	35.441	3.493
	DS_Petiole	5.027	1.971	11.128	21.558	7.061
	GS_Root	17.101	6.031	4.791	15.283	2.395
	GS_Trunk	4.547	1.427	3.744	17.812	2.419
	GS_Main stem	5.082	1.741	5.979	12.400	1.997
	IFS_Root	13.837	5.252	3.577	14.048	2.401
	IFS_Trunk	3.003	1.570	3.732	14.570	2.174

	IFS_Main stem	2.967	1.894	6.231	13.330	1.806
	IFS_Shoot	7.653	2.431	12.519	15.098	3.806
	IFS_Leaf	29.711	4.240	10.336	17.414	3.050
	IFS_Petiole	9.176	3.764	15.978	14.067	3.180
	IFS_Inflorescence	24.228	4.380	22.678	10.994	2.629
	EBS_Root	16.314	5.389	3.385	13.883	3.044
	EBS_Trunk	3.780	1.886	5.199	14.026	2.857
	EBS_Main stem	3.936	1.818	5.460	12.032	1.829
	EBS_Shoot	8.849	2.341	15.383	13.976	4.087
	EBS_Leaf	34.525	3.934	10.055	18.993	2.945
	EBS_Petiole	8.972	3.545	21.494	15.163	2.939
	EBS_Fruit	19.724	4.407	22.487	7.148	2.063
	SDS_Root	14.112	7.708	3.185	12.879	2.348
	SDS_Trunk	3.089	1.810	3.676	13.276	2.166
	SDS_Main stem	3.221	1.546	5.227	10.054	1.623
	SDS_Shoot	5.316	2.233	11.311	12.096	3.668
2021	SDS_Leaf	26.127	3.876	11.828	17.493	2.577
	SDS_Petiole	7.218	2.927	19.787	15.613	3.858
	SDS_Fruit	15.180	3.035	17.555	6.040	1.201
	VS_Root	16.016	8.030	4.057	15.368	2.543
	VS_Trunk	12.654	1.850	5.696	14.968	2.294
	VS_Main stem	3.549	1.647	5.637	7.063	1.314
	VS_Shoot	5.756	2.082	10.300	5.131	1.235
	VS_Leaf	18.246	2.725	9.137	19.575	2.978
	VS_Petiole	6.959	2.749	21.439	16.235	4.666
	VS_Fruit	6.693	1.854	15.965	7.905	2.391
	MS_Root	13.794	8.411	3.283	9.365	1.596
	MS_Trunk	7.803	1.221	3.481	20.992	2.415
	MS_Main stem	3.039	1.537	4.079	14.379	1.422
	MS_Shoot	3.852	1.992	7.431	5.480	1.069
	MS_Leaf	18.421	3.297	9.654	31.030	2.710
	MS_Petiole	5.790	3.246	17.535	21.892	6.399
	MS_Fruit	6.464	1.820	11.684	6.662	1.066
	DS_Root	14.369	6.212	3.106	10.253	1.437
	DS_Trunk	4.346	2.167	3.193	16.054	1.705
	DS_Main stem	4.887	2.020	4.389	9.384	1.509
	DS_Shoot	5.917	2.187	6.327	9.936	1.620
	DS_Leaf	8.502	2.807	7.153	37.074	3.709
	DS_Petiole	5.000	2.170	12.055	21.764	7.513
